# Bridging Structural Inhomogeneity to Functionality: Pair Distribution Function Methods for Functional Materials Development

**DOI:** 10.1002/advs.202003534

**Published:** 2021-01-22

**Authors:** He Zhu, Yalan Huang, Jincan Ren, Binghao Zhang, Yubin Ke, Alex K.‐Y. Jen, Qiang Zhang, Xun‐Li Wang, Qi Liu

**Affiliations:** ^1^ Department of Physics City University of Hong Kong Hong Kong 999077 P. R. China; ^2^ China Spallation Neutron Source Institute of High Energy Physics Chinese Academy of Science Dongguan 523000 P. R. China; ^3^ Department of Materials Science and Engineering City University of Hong Kong Hong Kong 999077 P. R. China; ^4^ Beijing Key Laboratory of Green Chemical Reaction Engineering and Technology Department of Chemical Engineering Tsinghua University Beijing 100084 P. R. China; ^5^ Shenzhen Research Institute City University of Hong Kong Shenzhen 518057 P. R. China

**Keywords:** local structure, neutron scattering, novel functional materials, pair distribution function, structural characterization, synchrotron X‐ray

## Abstract

The correlation between structure and function lies at the heart of materials science and engineering. Especially, modern functional materials usually contain inhomogeneities at an atomic level, endowing them with interesting properties regarding electrons, phonons, and magnetic moments. Over the past few decades, many of the key developments in functional materials have been driven by the rapid advances in short‐range crystallographic techniques. Among them, pair distribution function (PDF) technique, capable of utilizing the entire Bragg and diffuse scattering signals, stands out as a powerful tool for detecting local structure away from average. With the advent of synchrotron X‐rays, spallation neutrons, and advanced computing power, the PDF can quantitatively encode a local structure and in turn guide atomic‐scale engineering in the functional materials. Here, the PDF investigations in a range of functional materials are reviewed, including ferroelectrics/thermoelectrics, colossal magnetoresistance (CMR) magnets, high‐temperature superconductors (HTSC), quantum dots (QDs), nano‐catalysts, and energy storage materials, where the links between functions and structural inhomogeneities are prominent. For each application, a brief description of the structure‐function coupling will be given, followed by selected cases of PDF investigations. Before that, an overview of the theory, methodology, and unique power of the PDF method will be also presented.

## Graphical abstract

The pair distribution function (PDF) applications in a range of functional materials is reviewed. The outlines of principle, methodology, and unique powers of this technique have been firstly presented, followed by an introduction of its applications on selected functional material areas. Throughout this review, it has been attempted to emphasize the interplay between short‐range inhomogeneities and functionalities in novel functional materials.

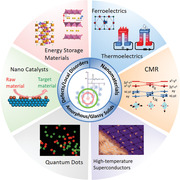

## 1 Introduction

Modern functional materials usually possess orders compromised with disorders. Nowadays, technological advances depend increasingly on novel functional materials engineered with complex structures at atomic and nanoscale levels.^[^ ^1^, ^2^, ^3^, ^4^, ^5^ ^]^ Typical examples are nano‐domains and domain walls that separate the coherent potentials (i.e., structure, electron, and spin) into fragments, which are the fundamental concept of interests in ferroelectrics, magnets, and superconductors.^[^ ^6^, ^7^, ^8^ ^]^ In addition, nano‐sized or nanostructured materials, on account of large specific surface area and limited dimension, are recognized as pathways for desirable catalytic and semiconducting (i.e., quantum dots (QDs)) functionalities.^[^ ^9^, ^10^, ^11^ ^]^ In the case of colossal magnetoresistance (CMR) magnets, the strong electron‐phonon coupling is mediated via a local Jahn‐Teller (J–T) distortion.^[^ ^12^ ^]^ This distortion is embedded in the nanoscale regions of metallic and insolating domains, whose balance is the key enabler to drive the coincident metal–insulator (M–I) transition.^[^ ^13^ ^]^ Furthermore, battery active materials are frequently engineered with defects and cation disorders for cathodes, or synthesized in nanoscale or amorphous phases for anodes.^[^ ^14^, ^15^ ^]^ An even more important fact is that the Li/Na insertion/extraction processes in the battery materials are actually inhomogeneous, so the key toward desired electrochemical performance lies in short‐range structural dynamics.^[^ ^16^ ^]^

To understand and design the functional materials in complex structures, it is crucial to gain knowledge of a short‐range structure deviated from the average. This is actually not a simple task since the conventional diffraction‐based crystallography on account of the Bragg's law is not developed for encoding aperiodic structures. Currently, the only methods of choice for probing short‐range structures include extended X‐ray absorption fine structure (EXAFS) and nuclear magnetic resonance (NMR), each of which owns their relative advantages.^[^ ^17^, ^18^ ^]^ Nevertheless, the spatial resolution limits of these techniques, such as ≈0.1 Å for the EXAFS, could be reached only for the first coordination shell around an atom, while the atomic interactions at greater distances cannot be distinguished with high accuracy.^[^ ^19^, ^20^ ^]^ On the other hand, the microscopic techniques, such as transmission electron microscopy (TEM) and atomic force microscopy (AFM), are capable of imaging the real‐space structure without the premise of long‐range ordering. However, the microscopic images are taken at a selected area, so it fails to give an accurate description if the structural complexity is heterogeneous over the materials. In such context, it is critical to provide statistical and precise descriptions of short‐range disorders embedding in the functional materials, for which the PDF method has demonstrated its success and wide applicability in the research area of functional materials.

The origin of the PDF method can be traced back in 1915 when the Debye scattering equation was first proposed,^[^ ^21^ ^]^ but for a long time this method has not been widely used until the recent 20 years when the techniques of synchrotron X‐rays and intensely pulsed neutrons were rapidly developed.^[^ ^22^ ^]^ These high‐energy and high‐flux beams allow for a greater reciprocal area to be detected (typically *Q*_max_ > 25–30 Å^−1^, discussed later), enabling a high spatial resolution of ≈0.01 Å that covers most concerns of structural complexities. As a result, the PDF technique, as a highly complementary detector to conventional bulk probes, could surely act as a powerful booster to the functional materials community. Herein we review the PDF applications in a range of functional materials, where the structural complexity plays a critical role in their functionalities. First, we will give an overview of principle, methodology, and unique powers of the PDF techniques, followed by the PDF applications on the selected areas of functional materials. Throughout this review, we attempt to emphasize the interplay between short‐range inhomogeneity and various kinds of functionalities, which are bridged by the PDF method.

## 2 Principles and Methods

Diffraction‐based crystallography has long been the basis for encoding the crystalline structure of a material. When X‐rays, neutrons or electrons are incident into crystals with perfect lattice periodicity, elastic scattering occurs and results in geometrical shadows—homocentric rings for poly‐crystals or spot array for single‐crystal—at well‐defined positions predictable by the Bragg's law:

(1) 2dsinθ=λ

which paints a clear picture of the diffraction feature correlating incident (or reflection) angle *θ*, interlayer spacing *d*(*hkl*), and radiation wavelength *λ*. The fascination of this equation is the power that the description of all atoms has been simplified to a small repetitive unit cell based on translational symmetry. By calculating *d*‐spacings and further quantitative analysis such as Rietveld refinement,^[^ ^23^ ^]^ an overview of average crystalline structure is provided, which is the key enabler for the revolution of materials science engineering over the past century.

**Figure 1:**
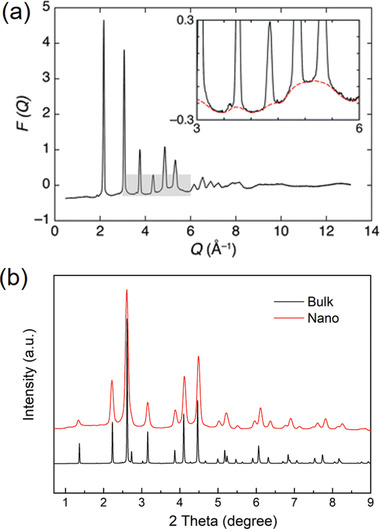
a) Illustration of diffraction and diffuse scattering signals spreading over *Q*‐space. The *F*(*Q*) presented here was normalized from the diffraction of AgBr. The inset emphasizes the diffuse signals associated with structural disorders. Reproduced with permission.^[^ ^19^ ^]^ Copyright 2011, Royal Society of Chemistry. b) Comparison of synchrotron XRD patterns of MnFe_2_O_4_ bulk and nanoparticles collected at 11‐ID‐C beamline (*λ* = 0.1173 Å).

The success of the Bragg's law relies critically on the assumption of perfect periodicity and long‐range lattice coherence. However, modern functional materials are usually engineered with defects and disorders, or synthesized in nanoscale or amorphous phases, in order to get desirable performance.^[^ ^24^, ^25^, ^26^ ^]^ In principle, once a structure locally deviates from the periodicity, the elastically scattered phonons would result in diffuse signals lying beneath and between the diffraction signals (**Figure** **1**a). In other word, these diffuse signals fingerprint the disorders. The traditional Bragg analysis deals with the diffraction and the diffuse signals separately. The structural information is extracted solely from the positions and shapes of the diffraction peaks, while the diffuse signals are subtracted as background. In this way, the translational symmetry of crystal structure is recovered to satisfy the Bragg's law, but meanwhile all the information regarding atomic disorders is abandoned. This problem could be even more prominent for nano‐crystalline without long‐range structural coherence.^[^ ^27^ ^]^ Due to the finite size effect of a nano‐crystalline, a remarkable line‐broadening of diffraction peaks is generated (Figure 1b). The conventional crystallography analysis describes these broadened patterns with the Scherrer equation,^[^ ^28^ ^]^ sometimes coupled with spherical harmonics for non‐*hkl*‐homogenous broadening‐shape.^[^ ^29^ ^]^ Yet, difficulties and errors are frequently emerged when atomic positions are considered, so only average lattice parameters of nanoparticles can be given even by fine analysis.^[^ ^30^ ^]^ Besides the finite size effect, nanoparticles also possess a high proportion of surface atoms, terminate with various *hkl*‐planes, and are highly disordered.^[^ ^31^ ^]^ All the above features contradict the premise of the Bragg's law and challenge the determination of nanoscale structures. On the other hand, glasses, increasingly applied as novel functional materials, show no crystalline periodicity but are not totally random like gases.^[^ ^32^ ^]^ For this non‐periodic family, the conventional diffraction‐based measurements only generate diffuse signal and provide no solution for structural determination. Actually, the structural description of glassy materials is still a great challenge in the fields of material science and solid‐state physics.

By contrast, the PDF method makes use of the entire diffraction and diffuse scattering signals, that is, total scattering signals, from a sample, hence providing two distinct views of a material: average orders and local disorders. This is surely a significant step forward especially for describing disordered structures at various length levels. In this section, we will describe (Section 2.1) how the PDF analysis can be used to characterize complex structures with total scatterings, (Section 2.2) what kind of structural information can be achieved in different conditions, and (Section 2.3) what are the unique advantages of total scattering PDF over conventional crystallography techniques.

### 2.1 Total Scattering Conversion

**Figure 2:**
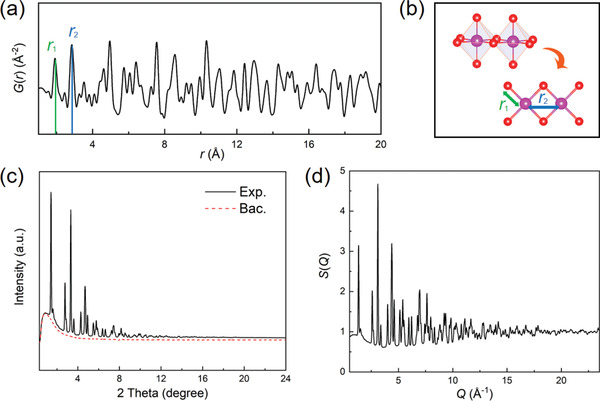
a) The atomic distribution of Li‐rich cathode material (Li_1.2_Ni_0.13_Co_0.13_Mn_0.54_O_2_) deriving PDF *G*(*r*) pattern. b) The schematic diagram atomic distance responsible for the first two shells. c) The raw powder diffraction data collected from 11‐ID‐C beamline, APS. The underneath red dashed line is the measured background for deduction. d) The total scattering function *S*(*Q*) data of Li‐rich material (*Q*_max_ = 23.5 Å^−1^).

The pair distribution function, that is, PDF, gives the weight probability of detecting any pair of atoms separated by a distance *r*—it establishes a distribution of interatomic distances in real space (**Figure** **2**a,b). To begin with, a few forms of PDF expressions are required to be clarified. The first one is defined as atomic pair distribution function*, g*(*r*):^[^ ^33^, ^34^, ^35^ ^]^

(2) $$g\left(r\right)=\frac{\rho \left(r\right)}{\rho_{o}}=\frac{1}{4\pi r^{2}N\rho_{o}}\sum_{i}\sum_{j\neq i}\delta \left(r-r_{ij}\right)$$

where *ρ*(*r*) and *ρ*_o_ are the local and average number densities of atoms, respectively, *r_ij_* refers to the distance between the *i*th and *j*th atoms in the system containing *N* atoms, and *δ* is the Dirac delta function whose integral equals to one only when *r* = *r_ij_*. This function of *g*(*r*) is the original definition of the PDF, but a reduced form, *G*(*r*), is more frequently used in practice:

(3) $$G\left(r\right)=4\pi r\left[\rho \left(r\right)-\rho_{o}\right]=4\pi r\rho_{o}\left[g\left(r\right)-1\right]$$

which makes it accessible to be transformed from experimental total scattering data (discussed later). Another important form of PDF is the radius pair distribution function, *R*(*r*):^[^ ^36^ ^]^

(4) $$R\left(r\right)=4\pi r^{2}\rho_{o}g\left(r\right)$$

This *R*(*r*) function enables a more clear physical picture to the PDF, since the exact number *N_r_* of atoms in a spherical shell with a thickness *dr* can be given by:

(5) $$N_{r}=\int R\left(r\right)dr$$

where *r* can be interpreted as the radius of the spherical shell.

Experimentally, the PDF can be converted from powder diffraction measurements using X‐rays, neutrons or electrons, considering the diffraction and diffuse signals equally. However, the overall intensity recorded out of a sample is more complicated and composed of several components in terms of different scattering processes:^[^ ^33^, ^37^ ^]^

(6) $$I_{\mathrm{tot}}=I_{e}\;+I_{\mathrm{ine}}+I_{m}+I_{\mathrm{bac}}$$

where *I*_e_ is the elastic (or coherent) scattering intensity, *I*_ine_ is the inelastic (or incoherent) scattering intensity, *I*_m_ is the multi‐scattering intensity, and *I*_bac_ is the background intensity. The intensities of diffraction and diffuse belong to the elastic scattering category (i.e., *I*_e_), which are the focus of PDF analysis carrying structural information. The inelastic scattering, arising from either Compton scattering of X‐rays or nuclear spin scattering of neutrons, could be eliminated by sensitive detectors or mathematics.^[^ ^37^, ^38^ ^]^ Note that the background referred here is generated from the scattering “outside” the sample (i.e., sample holder, sample environment, optical system, and so on). Obviously, this is very different from the background in diffraction‐based crystallography, which contains diffuse scattering from the sample. The standard procedure for the background subtraction is to collect *I*_bac_ just excluding the sample and deducting it from the raw data (Figure 2c). In addition, compared to X‐rays and neutrons, electrons show a more significant multi‐scattering effect due to their stronger interaction with atoms.^[^ ^39^, ^40^ ^]^ This results in an anomalous intensity of electron total scattering, hence extending electron diffraction to PDF is a great challenge.^[^ ^41^, ^42^ ^]^ Up to now, very few research works are based on electron PDF,^[^ ^43^, ^44^, ^45^ ^]^ which will not be an overview in this paper.

The total scattering function*, S*(*Q*), can be calculated from the elastic scattering intensity *I*_e_(*Q*) involving both diffraction and diffuse:

(7) $$S\;\left(Q\right)=1+\frac{I_{e}\left(Q\right)-\sum c_{i}{\left|f_{i}\left(Q\right)\right|}^{2}}{{\left|\sum c_{i}f_{i}\left(Q\right)\right|}^{2}}$$

where *c_i_* is the atomic concentration of the *i*th atom species and *f_i_* is the corresponding scattering factor for X‐rays or scattering length for neutrons. Here the variable *Q* is the amplitude of the momentum transfer **Q**, which can be defined by the difference between incident (**k_i_**) and scattered (**k_o_**) wave vectors for an elastic scattering:

(8) $$Q=2\pi \left(k_{i}-k_{o}\right)$$

(9) $$\left|Q\right|=Q=\frac{4\pi \sin\theta }{\lambda }\;$$

Briefly speaking, *Q* is a more generalized description of the scattering angle *θ*, taking account of the wavelength *λ* of X‐rays or neutrons. Since the diffuse scattering signal, which carries wealthy information about local disorders, is dominant at a high scattering angle, it is of particular importance to measure the total scattering data over a wide *Q* range—a large area in reciprocal space—to sensitively probe the local structures against the average.^[^ ^46^ ^]^ Nevertheless, as suggested by the Equation 9, the maximum *Q*, that is, *Q*_max_, that can be reached in experiments is limited by the wavelengths of applied X‐rays or neutrons. While the laboratory‐available Cu‐K*α* (*λ* = 1.538 Å) X‐ray source only gives *Q*_max_ ≈ 8 Å^−1^ and Mo‐K*α* (*λ* = 0.708 Å) gives *Q*_max_ ≈ 16 Å^−1^, the desirable *Q*_max_ value for accurate PDF analysis is 25 ≈ 30 Å^−1^ or higher. As a result, high‐energy synchrotron X‐ray or spallation neutron sources are the first choices, if not necessarily, for high‐resolution total scattering measurements.^[^ ^47^ ^]^ Apart from the limitation of wavelength *λ*, there are other factors, such as the detector boundaries and high‐*Q* noises, penalizing the *Q*_max_ that should be paid attention to.

Figure 2d shows the *S*(*Q*) pattern calculated from synchrotron X‐ray diffraction data of Li‐rich cathode material (i.e., Li_1.2_Ni_0.13_Co_0.13_Mn_0.54_O_2_, *Q*_max_ ≈ 23.5 Å^−1^). It appears different from the conventional powder diffraction shown in Figure 2b, not only because of the involvement of *Q*‐space; the total scattering function *S*(*Q*) has been divided by the square of atomic scattering factor <*f*(*Q*)>^2^ (Equation 7), which decreases at high‐*Q* region.^[^ ^48^ ^]^ As a result, the weak peaks or oscillations at high‐*Q*, mainly induced from the diffuse scattering signal, are amplified in *S*(*Q*) and become as equally important as the sharp Bragg peaks at low‐*Q*. The pair distribution function, *G*(*r*), can be converted from this high‐*Q*‐amplified *S*(*Q*) through a sine Fourier transformation:

(10) $$G\left(r\right)=\frac{2}{\pi }\int_{0}^{Q_{\max}}Q\left[S\left(Q\right)-1\right]\sin\left(Qr\right)dQ$$

which presents a histogram of atomic distances, or “bond lengths”, in the real space. In many cases, the term *Q*[*S*(*Q*)‐1] is also referred as *F*(*Q*), which further highlights the importance of high‐*Q* data.^[^ ^36^ ^]^ The power of this Fourier transform is to convert total scattering from reciprocal to real space, enabling a more intuitive view of the PDF data. Each PDF peak represents the probability of finding a pair of atoms between a given distance *r*. Obviously, the most straightforward way to analyze the local structure from PDF is to determine the peak positions in *G*(*r*), maybe coupled with a peak‐fitting process using a Lorentz or Gaussian function or their combination.^[^ ^37^ ^]^ Following the peak‐shifting evolution appears to be more efficient and intuitive especially for the continuous data series. Also, the heterogeneities in bonding environments, which is very important in some optical and thermoelectric materials, could be evaluated from the widths of the related PDF peaks.^[^ ^49^ ^]^ For a step forward, the coordination number between a pair of atoms could be estimated from the integrated area of the corresponding *G*(*r*) peak.^[^ ^19^ ^]^ Nevertheless, this operation is hard to proceed in many cases, due to the merging of PDF peaks and the background, as well as the weighted contribution from different types of atoms. Hence, computational analysis of the *G*(*r*) is required for local structure details and will be discussed later.

Practically, the whole total scattering conversion process, including the correction and calculation of *S*(*Q*), and the subsequent Fourier transform of *G*(*r*), could be implemented by programs. The widely recognized programs for the X‐ray PDF conversion are the PDFgetX3^[^ ^50^ ^]^ and recently developed xPDFsuite,^[^ ^51^ ^]^ while the PDFgetN is the generally used program to process neutron PDF data.^[^ ^52^ ^]^ At present, the programs are becoming increasingly automatic and user‐friendly to eliminate errors brought from the conversion. The most common error encountered in practice is the improper choice of the *Q*_max_ value. Although the total scattering function *S*(*Q*) generally extends to a wide *Q*‐space, the *Q*_max_ value employed in conversion is typically cut off below the experimental maximum to decrease the noises.^[^ ^53^ ^]^ However, a low‐*Q*_max_ conversion of *S*(*Q*) not only widens the *G*(*r*) peaks and reduces the real‐space resolution, but also produces strong ripples at a short distance (known as termination errors).^[^ ^54^ ^]^ Keeping the balance of *Q*_max_ is therefore important and requires a rich knowledge and experience in data analysis.

### 2.2 Computational Analysis

#### 2.2.1 Real‐Space Rietveld Method

The real‐space Rietveld method is a powerful and widely used approach to offer a quantitative solution of the local structure from the *G*(*r*) pattern.^[^ ^55^ ^]^ It is a full‐profile structural refinement similar to the well‐established Rietveld analysis in the diffraction‐based crystallography.^[^ ^1^ ^]^ The major similarity lies in the fact that they both are based on a certain “small‐box” unit‐cell model with identical structural parameters (i.e., lattice constant, atomic coordinate, atomic occupancy, and anisotropic thermal ellipsoid). Also, they share analogical calculating algorithm. However, as mentioned, the conventional Rietveld method only refines the Bragg peaks and treats the diffusion signals as background arbitrarily, hence resulting in an average structure of a given model. By contrast, the real‐space PDF Rietveld refinement deals with the total scatterings (i.e., diffraction and diffusion), which contains the local disorders deviating from the average structure.

The popular programs to perform the real‐space Rietveld refinement include PDFgui and DiffPy‐CMI.^[^ ^56^, ^57^ ^]^ The structural model is built with a unit cell containing fraction‐coordinated atoms, and then the structure parameters are refined for the best *G*(*r*) description using a least‐square method. To extract the local structural information, a larger supercell is frequently used instead of the crystallographic unit cell.^[^ ^58^, ^59^ ^]^ It is also a common strategy to adopt structural models with lower symmetry in order to release more degrees of freedom for possible atomic disorders.^[^ ^60^ ^]^ Furthermore, the *G*(*r*) pattern could be refined in separate Q‐space regions. While the refinement of *G*(*r*) at high‐*Q* region gives the long‐range average structure, the low‐Q refinement could provide insight into the local disorders in view of short‐range structure.^[^ ^61^, ^62^ ^]^ In this way, the departure of local structure from the average could be evaluated.

Compared to other computing techniques, the real‐space Rietveld analysis is the simplest method of choice that provides a first‐step glimpse of the local structure. Here we highlight the advantages of this method from the following aspects. First, the structural model extracted from the PDF refinement could be compared directly with that generated from the reciprocal‐space Bragg analysis, so these two approaches are complementary. This is usually the first stage in determining whether there is any short‐range disorder beyond the average structure. Second, the applied small‐box unit‐cell model, compared with the “big‐box” modeling discussed later, could be constructed easily, and the physical interpretations from the model are straightforward and visual. Moreover, some of the structural parameters, such as the atomic thermal factors and their ellipsoid anisotropies, that related heavily to the local structure are considered to be more precisely determined from the real‐space than reciprocal space analysis.^[^ ^33^ ^]^ As a consequence, the real‐space Rietveld method is widely accepted and applied in analyzing PDF and will be increasingly important in studying novel functional materials that tend to be more disordered.

#### 2.2.2 Reverse Monte Carlo (RMC) Simulation

**Figure 3:**
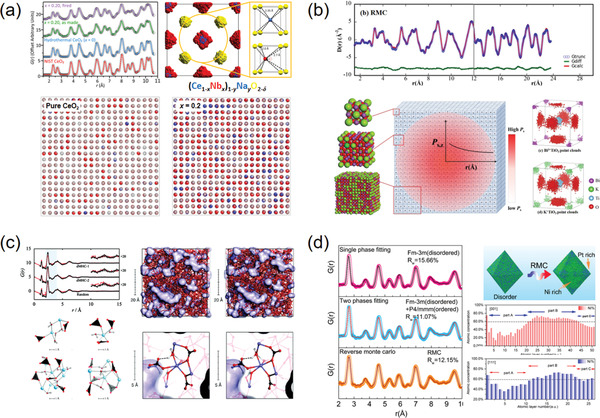
a) RMC models and simulations of neutron total scattering from Nb‐doped CeO_2_. A 10 × 10 × 10 supercell was built as the initial model and the fitting result of atomic displacements was conformed into a single fluorite unit cell. Reproduced with permission.^[^ ^71^ ^]^ Copyright 2018, American Chemical Society. b) RMC fitting results of synchrotron X‐ray total scattering data for Bi_0.5_K_0.5_TiO_3_ ferroelectric perovskite using a 12 × 12 × 12 supercell. The atomic displacement distributions were folded into the *P*4*mm* unit cells. Reproduced with permission.^[^ ^72^ ^]^ Copyright 2017, American Chemical Society. c) RMC models and simulation results of X‐ray total scattering data for a clear view of the nanoporous channels and short‐ and medium‐range orders in the amorphous hydrated calcium carbonate. Reproduced with permission.^[^ ^69^ ^]^ Copyright 2010, American Chemical Society. d) RMC fitting results of synchrotron X‐ray PDF for determining the component segregations in a single Pt_41_Ni_59_ alloy nanoparticle. Reproduced with permission.^[^ ^73^ ^]^ Copyright 2017, American Chemical Society.

The real‐space Rietveld method is incapable of recognizing unknown structures, as it depends on a certain unit cell with specific crystallographic symmetry. Also, it is impossible to simply describe a highly disordered structure, such as heterogeneous gradient systems or amorphous phases, with a repeating small‐box unit. For these situations, an alternative solution to analyze PDF without the small‐box and symmetric constraints is necessarily required for encoding the complex structures. The reverse Monte Carlo (RMC) technique is developed based on an opposite philosophy to the Rietveld—it puts a large ensemble of atoms (hundreds to thousands) into a 3D big‐box configuration and randomly arranges them to search for a good fit of the experimental *G*(*r*) data.^[^ ^63^, ^64^, ^65^ ^]^ The purpose here is to create a sufficiently large space for describing disorders and local structural correlations within the initial model. Clearly, the scheme of RMC modeling is pellucid, and is attractive to not only amorphous materials (i.e., silicon, metallic glasses, and so on),^[^ ^66^, ^67^, ^68^, ^69^ ^]^ but also crystals with significant disorders in structure, site occupancy, electrons, spin, and atomic thermal vibrations.^[^ ^70^, ^71^, ^72^, ^73^ ^]^ A few examples of the RMC modeling are shown in **Figure** **3**.

The original RMC code was developed for extracting the atomic structures of liquids and net‐work glasses from the diffuse signals, which has given birth to several RMC programs like DISCUS and RMC++.^[^ ^74^, ^75^ ^]^ For a further step, the program RMCProfile has extended the RMC technique approaching to the disordered crystalline materials, implemented by fitting a separate profile of Bragg diffraction, that is, F(Q), along with the *G*(*r*) function.^[^ ^76^ ^]^ For a typical procedure, the big‐box configuration is constructed with the periodic conditions, and the atoms in the box are allowed to move randomly in order to produce a structural model consistent with the experimental data. To avoid unphysical solutions, it is critical to impose constraints (e.g., bond lengths, bond angles, coordination numbers, and so on) into the RMC refinements as appropriate.^[^ ^77^ ^]^ Note that the final target of the RMC simulation is to search for rules from the random (a structure output at random is meaningless to the crystallography!). For the crystalline materials, this is usually accomplished by folding all the atomic displacements into their crystallographic unit cells to map the “atomic clouds” (Figure 3a,b).^[^ ^71^, ^72^ ^]^ In the cases of amorphous materials, the ligand environments within near r distances could be also summarized from the simulated structural models (Figure 3c).^[^ ^69^ ^]^

At present, the combination of total scattering and RMC modeling has been recognized its power and applied extensively in the fields of amorphous materials and disordered crystalline materials. However, the importance of RMC in studying nano‐materials might be undervalued. In fact, the RMC method has been manifested as capable of modeling a nanoparticle with tens to a hundred thousand closely‐packed atoms in a larger box, where each nanoparticle is isolated properly considering the periodic conditions (Figure 3d).^[^ ^73^ ^]^ In this way, the surface‐to‐bulk inhomogeneities (i.e., surface reconstructions, segregations of elements and phases, and so on) in dimension‐limited systems could be simulated. Recently, some algorithmic progress has been made to correct the errors brought from removing the periodic boundary condition when the low‐dimensional box is built.^[^ ^78^ ^]^ As this correction will be made available via the RMCProfile program, the RMC method would play a more important role in clarifying the inhomogeneities in low‐dimensional nano‐systems.

#### 2.2.3 DFT/Molecular Dynamics Coupling

The RMC simulation has enabled a pathway to encoding the highly disordered materials with a 3D atomic configuration. However, it is always a great challenge to properly build the starting models. For the amorphous and non‐periodic materials, especially, this big box is actually more like a “black box” with unknown structure, which contains a large ensemble of atoms arranging randomly regardless of energetics and thermodynamics. Even if the model is constrained with given bonding environments, it is insufficient, complex, and may enroll errors, raising a question as to whether the RMC method could be extended to widespread researches of disordered materials.

In such a context, ab initio theoretical techniques, such as density functional theory (DFT), molecular dynamics (MD), and their combination (DF‐MD), have been frequently used to predict the feasible structure of the starting model. For example, the heterogeneous structure of SiO containing an amorphous Si‐SiO_2_ hybrid could be revealed by the X‐ray total scattering coupling RMC, where MD simulations were applied to reduce the total energy of the starting model.^[^ ^79^ ^]^ Analogously, the DFT‐relaxed geometric models of La‐ and Mn‐substituted BiFeO_3_, compared to the models constrained only by crystallographic symmetry, were demonstrated more successfully in the descriptions of *G*(*r*) patterns.^[^ ^80^ ^]^ Furthermore, by coupling DF‐MD with RMC, the total scattering data of amorphous Sb‐Te‐based alloy and also hydrated SnO_2_ surface could be reproduced with efficiency.^[^ ^81^, ^82^ ^]^ One common practical application of this strategy is to construct and optimize the 3D models of metallic nanoparticles such as Au‐, Pt‐, and Pd‐based nanoalloys, normally with MD corrected by the Sutton–Chen potential.^[^ ^83^, ^84^, ^85^ ^]^ For all the above cases, the processes of the theoretical prediction and RMC modeling are isolated, which means the empirical constraints are still required during the RMC reconstruction. To minimize the uncertainty of RMC modeling, a DFT‐RMC iterative process has been developed, in which the atomic model could be constrained and relaxed by DFT throughout the data‐driven modeling.^[^ ^86^ ^]^ Using this method, the final structure of amorphous kaolinite that is both experimentally reasonable and energetically feasible could be accessible.^[^ ^87^ ^]^

Undoubtedly, both RMC and ab initio computational techniques are extremely powerful but meanwhile inevitably labor intensive. As a result, these methods are cautioned to be applied at the very beginning of the PDF analysis, particularly in the cases of materials with known average structures. The small‐box Rietveld approach should be the first‐step method of choice, if the guidance of *G*(*r*) peak shifting is not clear or sufficient enough to get structural information. Nevertheless, as the today's functional materials are increasingly embedded with disorders, the RMC‐based total scattering techniques will surely play a more important role in unraveling the structural origins of properties and related functionalities.

### 2.3 Power of PDF Methods

#### 2.3.1 Defects and Local Disorders

Lattice defects, such as point defects, dislocations, grain boundaries, and so on, strongly affect the intrinsic properties of materials through a sudden perturbation of crystallographic coherence. Modern functional materials are usually engineered with defects, as they could act as a regulator for acquiring desired performance.^[^ ^88^, ^89^, ^90^ ^]^ In general, the crystal structure within a defective area is heavily distorted, and may be reconstructed into domains or defect clusters for a lowered total systematic energy.^[^ ^91^ ^]^ Nevertheless, a full and accurate description of this defective structure is experimentally difficult and still awaited for many functionalized systems. The conventional diffraction‐based crystallography gives only long‐range average structure and is cautioned for probing defective structures, because the weak diffuse signals generated from defects and local disorders are disregarded. The recent development of atomic‐resolved STEM technique has enabled an approach to visualizing the defects and related disorders,^[^ ^92^ ^]^ yet it only provides the structural information within a selective area, and a statistical result regarding the defective structure is still needed.

**Figure 4:**
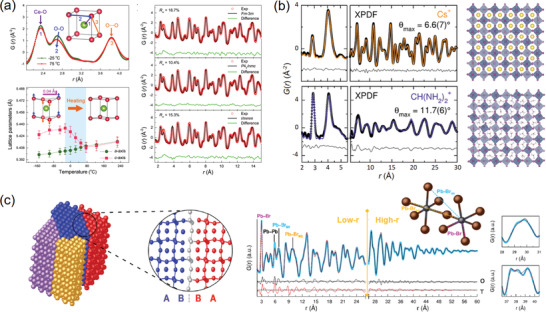
a) The small‐box neutron PDF refinements of nanosized ceria, revealing a tetragonal phase and a tetragonal‐to‐cubic phase transition induced by oxygen vacancies. Reproduced under the terms of CC‐BY Creative Commons Attribution 4.0 International License.^[^ ^60^ ^]^ Copyright 2018, The Authors, published by Springer Nature. b) The X‐ray PDF refinements of Cs_2_SnI_6_ and (CH(NH_2_)_2_)_2_SnI_6_ with 3 × 3 × 3 supercell structure models. In these models, the rigid octahedral units were randomly rotated with a maximum rotating angle *θ*_max_. Reproduced with permission.^[^ ^104^ ^]^ Copyright 2017, American Chemical Society. c) The X‐ray PDF refinements of CsPbBr_3_ nanocrystals with a PbBr_6_‐octahedron tilted structure model. The tilted domains are joint with twin boundaries. Reproduced with permission.^[^ ^105^ ^]^ Copyright 2017, American Chemical Society.

With the consideration of both diffraction and diffuse signals, the PDF technique is one of the few available pathways toward local disorders and defects. For a typical example, CeO_2_ has conventionally been thought to own a cubic fluorite structure (*Fm*‐3*m*) even under a highly defective condition, but this idea has recently been challenged by neutron PDF investigations, which showed that the existence of oxygen vacancies could induce a tetragonal (*P*4_2_/*nmc*) structure and drive a reversible tetragonal‐to‐cubic phase transition in a temperature range of −25 ≈ 75 °C (**Figure** **4**a).^[^ ^60^ ^]^ In another case, the monoclinic‐to‐cubic phase transition occurred in La_2_Mo_2_O_9_ oxygen‐ion conductor was demonstrated to be driven by the dynamical distribution of oxygen defects, evidenced by neutron PDF analysis.^[^ ^93^ ^]^ Additionally, the impact of anion vacancies on the local structure of Fe‐based oxyfluoride electrode material was also investigated by X‐ray total scattering combined with DFT.^[^ ^94^ ^]^ The results demonstrated that the formation of oxygen vacancies is coupled with a spontaneous rearrangement of the local structure, hence triggering a remarkable enhancement in electronic transport and Li insertion processes during battery reactions. Furthermore, the unique power of PDF method in probing cation/anion defects and the derived local distortions has been manifested in a variety of functional materials such as UO_2_ nuclear fuel,^[^ ^95^ ^]^ defect‐rich thermoelectric Eu_2_ZnSb_2‐x_Bi_x_,^[^ ^96^ ^]^ high‐entropy alloys,^[^ ^97^ ^]^ and so on.

On the other hand, the stacking faults, capable of shaping band structures and gap energies, play an important role in the performance of semiconductors, catalysts, and optical materials.^[^ ^98^ ^]^ This special type of planar defect could be also determined by fine analysis of total scattering. By refining the *G*(*r*) pattern with a DFT‐relaxed stacking model, the stacking faults could be clearly identified within the nanodomains of *γ*‐Al_2_O_3_ matrix, providing a new insight into its catalytic functionality.^[^ ^99^ ^]^ In addition, the strain‐driven stacking faults in CdSe/CdS core‐shell nanorods could be also probed by X‐ray PDF along with STEM experiments.^[^ ^100^ ^]^ Recently, an *R*‐space PDF analytical method has been developed, dedicated to encoding the stacking faults in close‐packed metals, which would be helpful to the researches of metallic nanoalloys.^[^ ^101^ ^]^

In practice, the small‐box Rietveld‐like refinement is an efficient way to reconstruct the defective structure from total scattering data. In order to depict this disordered structure, the applied unit cell model is often built in a lowered symmetry, so that the roles of defects could be concluded as local distortions.^[^ ^60^, ^102^, ^103^ ^]^ Meanwhile, a coupling strategy is to construct a larger supercell, instead of the smaller crystallographic one, to tolerate more inhomogeneity in the whole system. As shown in Figure 4b, the X‐ray PDF modeling of vacancy‐ordered *A*_2_SnI_6_, where *A* = Cs^+^ and CH(NH_2_)_2_^+^, were carried out by constructing 3 × 3 × 3 supercells, where random rotations of rigid octahedral units were introduced with a maximum rotating angle *θ*_max_. The results of PDF modeling revealed that the replacement of Cs^+^ with CH(NH_2_)_2_^+^ in *A*‐site of perovskite *A*_2_SnI_6_ could induce a more disordered structure with a larger *θ*_max_ value, holding implications for the electron‐phonon interactions and related optoelectronic applications.^[^ ^104^ ^]^ Another way of probing the defect‐induced local disorders from PDF analysis is to separately refine the low*‐r* and high*‐r* regions in the *G*(*r*) pattern. The low*‐r* region is more sensitive to the defects and local disorders, as it enrolls a greater proportion of diffuse signals compared to the high‐*r* region. Using this method, the nanotwin structures in CsPbX_3_ (X = Cl, Br, I) nanocrystals was able to be clarified.^[^ ^105^ ^]^ It was shown that low‐*r* pattern could be refined with PbX_6_ octahedron‐tilted orthorhombic subdomains, while the high‐*r* pattern could be matched with the tetragonal phase (Figure 4c). These tilted subdomains are hinged through the network of twin boundaries. Lastly, a more realistic picture of a defective structure could be drawn by the big‐box RMC analysis of total scattering. The distribution of defects and also the reconstructed coordination around defects could be statistically mapped by the RMC method.^[^ ^106^, ^107^ ^]^

#### 2.3.2 Nanomaterials

The finite‐size effects have enabled nano‐sized materials with peculiar chemical and physical features, and endowed functional nanodevices with great potentials.^[^ ^31^ ^]^ In the past few decades, tremendous research and development efforts have been devoted to miniaturize the devices for potable capability and novel functionality. The success of these efforts depends on a robust understanding of nanomaterial structures especially at atomic scales. As mentioned above, the conventional diffraction‐based techniques provide only lattice constants and an average symmetry of nanocrystallines, while the atomic structural details, such as coordinations of atoms, surface reconstructions, anisotropic thermal ellipsoids, and so on, would be hidden behind the broadened and overlapped Bragg peaks at the high‐*Q* region. Moreover, the materials in nanoscale are usually heterogeneous in compositions and phases, which introduces additional complexities into the nanostructures.

**Figure 5:**
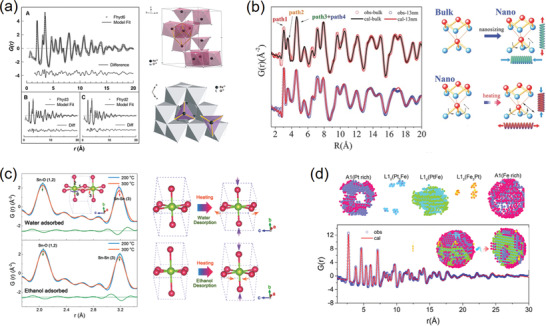
a) The polyhedral‐interconnected unit cell of ferrihydrite mineral resulted from the small‐box PDF refinements. Reproduced with permission.^[^ ^112^ ^]^ Copyright 2007, American Association for the Advancement of Science. b) The surface structural change upon nanosizing and heating in 13 nm Bi particles extracted from the PDF real‐space refinements. Reproduced under the terms of CC‐BY Creative Commons Attribution 4.0 International License.^[^ ^115^ ^]^ Copyright 2016, The Authors. Published by Wiley‐VCH. c) The surface structural evolutions upon water and ethanol desorption upon heating achieved from the PDF refinements. Reproduced with permission.^[^ ^119^ ^]^ Copyright 2018, Wiley‐VCH. d) The 3D diagram of the phase segregation in 3.5 nm PtFe alloy particles stripped from the PDF‐RMC simulated model. Reproduced with permission.^[^ ^123^ ^]^ Copyright 2018, American Chemical Society.

The commonly used methods for probing nanostructures include EXAFS and PDF. Among them, the EXAFS technique is capable of probing the coordination environments around one certain atom. Currently, many of the current works adopt the EXAFS method to study nanomaterials, taking advantage of its elemental sensitivity. Especially, the polarization‐dependent X‐ray spectroscopy technique is capable of probing the orientational effects on surfaces, which is also a feasible approach to detect the local structure of nanomaterials.^[^ ^108^, ^109^ ^]^ However, as mentioned above, the high spatial resolution of the EXAFS (≈0.1 Å) could be achievable only for the first coordination shell, while the atomic interactions at greater distances cannot be determined with high accuracy. This hinders the wide applicability of the EXAFS method. On the other hand, the PDF method has been widely applied and demonstrated to be brilliantly successful in probing the structures of nanomaterials. This could be attributed to its advantageous feature for describing finite‐size nanocrystals—it summarizes the entire nanoscale system from the nearest‐neighbor bonds up to the furthest‐distance atom pairs.^[^ ^110^ ^]^ In view of this, the confined size of a nanoscale material can be estimated from the termination of *G*(*r*) fluctuation.^[^ ^111^ ^]^ One straightforward motivation for studying nanomaterials with PDF is to obtain reliable atomic coordinations for the *G*(*r*) refinements, so that the averaged atomic structures, such as bond lengths and bond angles, could be extracted for further investigation. In this respect, the structure clarification of ferrihydrite is a classic example. Although the ferrihydrite is ubiquitous in nature and widely applied as an industrial sorbent, there has been no consensus regarding its crystal structure for a long time, because this mineral exists in the form of a nanocrystalline (<10 nm in general). By means of real‐space PDF modeling, the atomic structure of ferrihydrite was uncovered for the first time.^[^ ^112^ ^]^ This structure adopts a single hexagonal *P*6_3_ *mc* phase, consisting of 20% tetragonal‐ and 80% octahedral‐coordinated Fe(III) ions (**Figure** **5**a), which breaks the typically believed multiphase structure model for ferrihydrite. Later, the aging structure of ferrihydrite, as well as its relation to the magnetic structure was also revealed by X‐ray PDF analysis.^[^ ^113^ ^]^ In a recent case, the structure of nano‐sized MoO_2_, which is known to differ from the bulk distorted rutile phase, was initially determined by the small‐box refinements of X‐ray PDF data.^[^ ^114^ ^]^ For the nanoparticles larger than 40 nm, the overall rutile structure could be retained, while the ultrafine nanoparticles (<5 nm) could be described with either Magnéli or De Wolff structural model with rich defects. This size‐induced structural change could offer a possible explanation of the better electrochemical performance of nanoscale MoO_2_ anodes for Li‐ion batteries. These examples illustrate the strength of PDF in probing nanostructures, and most of them are performed with Rietveld‐like refinements. It is worth noting that this PDF Rietveld method should not be just regarded as a diffraction refinement in real space, as it enlists diffuse signals that are sensitive to the short‐range coherence, such as surface structures, heterogeneous nanoregions, and so on. With this in mind, the atomic structures of nanomaterials extracted from diffraction should be cautiously used for further analysis, even in the case that the well‐defined Bragg peaks are generated.

For nanocrystals, the PDF analysis averages the structures from the external surface (or interface) to the internal core. Since the core atoms in the nanocrystals theoretically possess nearly the same coordination environments with those in the bulk, it is rational to speculate that the difference between the nano and bulk structures given by PDF is derived from the surface reconstructions. As a result, the PDF technique could act as a surface detector of nanomaterials when comparing and extracting the bulk structure out of it. For example, the surface distortions in Bi nanoparticles and the related thermal behaviors were successfully determined using X‐ray PDF analysis.^[^ ^115^ ^]^ By comparing the PDF‐derived structures of bulk and nanoparticles, the bond lengths and bond angles on the corrugated layer surface are revealed to change remarkably, which affects the bending acoustic waves and induces an uniaxial negative thermal expansion (NTE) in the nanosized bismuth (Figure 5b). Likewise, the local octahedral distortions near the twin boundaries of SnO_2_ nanowires could be also determined by the PDF analysis.^[^ ^116^ ^]^

On the other hand, the surface of a nanoparticle is rich in dangling bonds that make it easy to adsorb small molecules (i.e., H_2_O, ethanol, and so on). This has led to a variety of important applications such as TiO_2_ photocatalysts and SnO_2_ gas sensors, where knowledge of surface structural changes upon adsorption is a fundamental issue.^[^ ^117^ ^]^ In this respect, the PDF method enables a feasible solution, which could be realized by comparing the surface structures with and without adsorbates from the PDF analysis. For example, the surface structures of anatase‐phase TiO_2_ nanoparticles upon water adsorption were clarified by the X‐ray PDF refinements.^[^ ^118^ ^]^ It is found that the dissociative adsorption of water, which is the first step of photocatalytic water splitting, takes place mainly on highly‐active {001} facets, leading to a significant modification of the Ti—O—Ti bonding environment. Such a (*hkl*)‐dependent change in the surface structure is demonstrated to greatly impact the thermal expansion behavior of TiO_2_ nanoparticles. For the SnO_2_ ethanol gas sensors, the sensing sensitivity drops largely from a fundamental study to real‐life application, which is a result of the ambient interference especially the humidity. However, understanding this phenomenon is challenging and intricate, as it involves the adsorptions of water, ethanol, and their mixture on the surface. The X‐ray PDF analysis provides a new insight into the cross‐sensitivity of SnO_2_ ethanol sensor to humidity, indicating that the performance of ethanol sensors is strongly coupled with the surface structures upon adsorptions.^[^ ^119^ ^]^ During the sensing process, the SnO_2_ surface is adsorbed with water and ethanol simultaneously, so their independent effect could not be achieved simply from the PDF data of clean SnO_2_. In this case, the adsorbed nanoparticles were heated for the desorptions of water and ethanol at distinct temperatures, and thus the relative roles of water and ethanol could be deduced from the thermal lattice evolutions (Figure 5c).

Moreover, nanosized materials frequently undergo segregations of strains, phases or chemical compositions, which could be driven from either the surface effects or the synthetic processes.^[^ ^120^, ^121^ ^]^ These heterogeneous nanodomains are proven to be very important to functional properties such as magnetic response and catalytic activity, yet depicting a clear picture of the segregations has always been a great challenge.^[^ ^122^ ^]^ The PDF coupled with RMC technique is one of the few available approaches toward segregated domains in nanomaterials. For example, the local strains and the related A1‐L1_0_‐L1_2_ phase segregations in PtFe nanoalloys are deciphered with PDF‐RMC modeling.^[^ ^123^ ^]^ From the disassembly of the simulated structure, the surface of the prepared PtFe nanoparticle shows a disordered A1 phase, while the core lattice exhibits mainly a Pt/Fe stacking L1_0_ structure (Figure 5d). The stretching strains induced from the lattice mismatch between these two phases could affect the overall magnetic property and thermal behavior of the PtFe nanoalloys. In the case of PdAu nanoparticles, the RMC modeling was carried out to investigate the compositional and structural heterogeneity.^[^ ^124^ ^]^ The RMC‐simulated results indicate an enrichment of Pd on the surface, which is synthetically directed by prior binding of the Pd‐containing peptide. Thus, the PDF‐RMC simulation has enabled an opportunity for the rational design of the surface chemistry, structure, and strains in bimetallic nanoalloys for a desirable catalytic activity.

#### 2.3.3 Amorphous or Glassy Solids

An amorphous solid is a condensed matter that lacks the long‐range order. It is sometimes synonymous with the terms of “glass” or “glassy solid”, but is strictly a glass that should undergo a glass transition.^[^ ^125^ ^]^ The classic amorphous solids include metallic glasses, polymers, vitreous silica, porous carbon, etc., and any amorphous precursor before crystallization. For a long time, amorphous materials were rarely employed as functional materials as they are complex and unpredictable. However, a clear trend is emerging today, placing the amorphous materials at the forefront of research in broad areas covering catalysts, sensors, superconductors, energy storage devices, and so on.^[^ ^124^, ^125^, ^126^, ^127^, ^128^ ^]^ In principle, the structures of the amorphous materials are nonperiodic yet not totally random—they are composed of interconnected unit clusters that could be similar with their crystalline phases.^[^ ^129^ ^]^ Clearly, the conventional diffraction‐based crystallography provides no solution for such complex structures, and it is also not wise to specify all the atomic positions in an amorphous system from microscopic techniques, even though the resolution is sufficiently high enough to reach an atomic scale.

**Figure 6:**
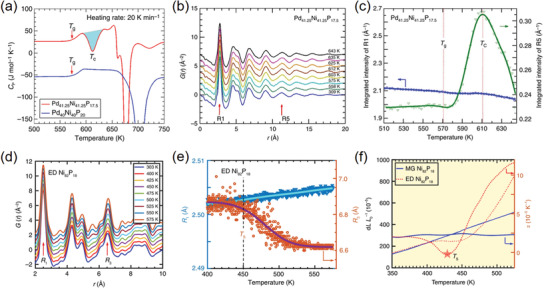
a) The DSC anomaly in Pd_41.25_Ni_41.25_P_17.5_ at *T*_C_ ≈612 K, while no anomaly could be observed in Pd_40_Ni_40_P_20_. b) Reduced *G*(*r*) patterns collected from the Pd_41.25_Ni_41.25_P_17.5_ alloy at different temperatures. c) Integrated intensity of from the R1 to R5 shells in the *G*(*r*) patterns. Reproduced under the terms of CC‐BY Creative Commons Attribution 4.0 International License.^[^ ^132^ ^]^ Copyright 2017, The Authors. Published by Springer Nature. d) Reduced *G*(*r*) patterns collected from ED Ni_82_P_18_ alloy at various temperatures. e) The temperature‐dependent peak positions in the variable‐temperature *G*(*r*) patterns of the ED Ni_82_P_18_ alloy. f) Anomalous thermal expansion behavior in the ED Ni_82_P_18_ alloy, in comparison with the metallic glass (MG) Ni_82_P_18_ alloy with normal positive thermal expansion behavior. Reproduced under the terms of CC‐BY Creative Commons Attribution 4.0 International License.^[^ ^133^ ^]^ Copyright 2019, The Authors. Published by Springer Nature.

In fact, the total scattering technique is initially developed for tackling this “amorphous problem”. Since the unit clusters possess certain coordination environments, they could produce sharp peaks within the short *r*‐distance of the *G*(*r*) patterns. As a result, many efforts have been devoted to reproduce the local structures of amorphous solids from the PDF peak positions, where the first several shells could represent the neighbor bonding and global packing of the unit clusters, respectively.^[^ ^130^, ^131^ ^]^ As a typical example, metallic glasses are amorphous metals (usually alloys) that exhibit unique properties with respect to their crystalline counterparts. However, there exists a long‐standing mystery in a number of metallic glasses, whose differential scanning calorimetry (DSC) curves show an anomalous exothermal peak between the glass and crystallization temperatures (**Figure** **6**a). This indicates an unknown amorphous phase transition during the heating process. To address this issue, Lan et al. carried out a series of PDF measurements on Pd_41.25_Ni_41.25_P_17.5_ alloy, in order to gain atomistic insights into the structural evolutions at different temperatures (Figure 6b).^[^ ^132^ ^]^ The results reveal a hidden amorphous phase in this alloy, and therefore the anomaly in the DSC curve is resulted from a polyamorphous phase transition at the corresponding temperature. This phase transition could be regarded as the rearrangement of the local clusters in the medium range (up to ≈18 Å), which is fingerprinted by integrated *G*(*r*) intensity from the first (R1) to the fifth (R5) shells (Figure 6c). In addition, it has recently been found that this medium‐range packing could be engineered with electrodeposition (ED).^[^ ^133^ ^]^ The PDF patterns of the ED Ni_82_P_18_ alloy could visualize both short‐range and medium‐range shells from the first (R_1_) and the third (R_3_) peaks, providing key evidence of this medium‐range engineering (Figure 6d). Remarkably, it has been shown that the nearest R_1_ expands while the R_3_ contracts upon heating, giving rise to an anomalous thermal expansion behavior in the ED alloy (Figure 6e,f). This suggests that engineering the packing arrangements of unit clusters could pave a feasible way to tailor a thermal expansion property in metallic glasses. Additionally, a variety of mechanical processes in metallic glasses, such as elastic deformation,^[^ ^134^ ^]^ high‐pressure torsion,^[^ ^142^ ^]^ and so on, could be encoded by following the peak evolutions in the *G*(*r*) pattern series.

The clarification of amorphous‐to‐crystalline dynamics is another important application of PDF in this area. For example, the crystallizing process of amorphous zeolites was investigated by identifying the first few shells in the *G*(*r*) patterns.^[^ ^135^ ^]^ The results reveal a clear picture of the local structural evolution during the induction period of crystalline, which involves a condensation process of aluminosilicate units and a subsequent construction of a large‐ring structure. In another case, the nucleation of layered titanates, which are the precursors of anatase or rutile TiO_2_ in wet‐chemical synthesis, was studied by in situ X‐ray PDF analysis during the hydrothermal reaction.^[^ ^136^ ^]^ These intermediate layers present lepidocrocite‐type structure along the *ab* plane, so the generated *G*(*r*) patterns could be described well with a small‐box nanosheet model. The PDF results show that the lepidocrocite‐layered titanates are formed directly in the alkaline solution with cluster sizes <0.7 nm, while the subsequent thermal treatment connects these pre‐structures into periodic 2D layers. For the reverse procedure, the amorphization of monoclinic Nb_2_O_5_ under high pressure could be also illuminated by PDF measurements.^[^ ^137^ ^]^ By following the peak evolutions at variable pressures, the amorphization is revealed to be closely related to the breakdown of long‐range polyhedral chains, whereas the local unit blocks of edge‐sharing NbO_6_ octahedra and pentagonal bipyramids could be maintained during this procedure.

Typically, the PDF data collected from an amorphous system is difficult to be analyzed computationally. The barrier lies in the fact that it is impossible to describe a glassy solid with a repeating small‐box unit. With regard to this, the large‐box RMC simulation could be the only method of choice to feedback an amorphous structural model from the PDF data. The first RMC modeling of amorphous SiO_2_ was performed in 1990,^[^ ^138^ ^]^ and since then the algorithm has been optimized significantly, capable of combining MD and DFT, and triggered the RMC studies in a variety of amorphous systems such as metallic glasses,^[^ ^139^ ^]^ hydrated complexes,^[^ ^69^ ^]^ SiO*_x_*,^[^ ^79^ ^]^ amorphous MOFs,^[^ ^140^ ^]^ porous carbon,^[^ ^141^ ^]^ and so on. Despite the above success, nonetheless, this method is frequently questioned as to whether the PDF data itself can carry such a huge information about every random atomic position in an amorphous system, and whether the retrieved chemical or topological results are model‐independent. These, up to now, are still open questions, but doubts maybe gradually eliminate by more exposure of successful cases and further improvements in RMC algorithms.

## 3 Applications in Functional Materials

In this section, we will review the PDF‐related applications in functional materials, placing emphasis on how the structural inhomogeneities play roles in their functionalities, and how the PDF method promotes the developments of these materials. For each application, we will begin with a brief introduction of the links between functions and structural, electronic or magnetic inhomogeneities, followed by selected cases of PDF investigations and possible operando PDF measurements applied in temperature, electric and magnetic fields.

### 3.1 Ferroelectrics/Thermoelectrics

#### 3.1.1 Ferroelectrics

A ferroelectric material is identified by the occurrence of spontaneous polarization (*P_S_*) that is switchable in response to an external electric field.^[^ ^143^ ^]^ This unique feature has enabled it to be functionalized and widely applied in piezoelectric transducers, non‐volatile memories, fatigue‐free capacitors and so on.^[^ ^144^, ^145^, ^146^ ^]^ Theoretically, the properties of ferroelectricity are presented only in crystalline materials with asymmetric groups, where the positive charge center, typically in MO_6_ (M = metal) octahedra, is slightly displaced away from its axis‐symmetric position.^[^ ^147^ ^]^ As a result, one central topic in the research field of ferroelectrics is the crystallography‐polarization coupling, which is also critically challenging due to its complex structural concepts including off‐center distortions, different‐orientated domains, polar nanoregions (PNRs) and so on. In addition, modern ferroelectric materials are usually made of nanomaterials or engineered with dopants and solid solutions, which also introduce structural complexities. With this regard, the PDF method could play an important role in studying the ferroelectric materials and related physics.

**Figure 7:**
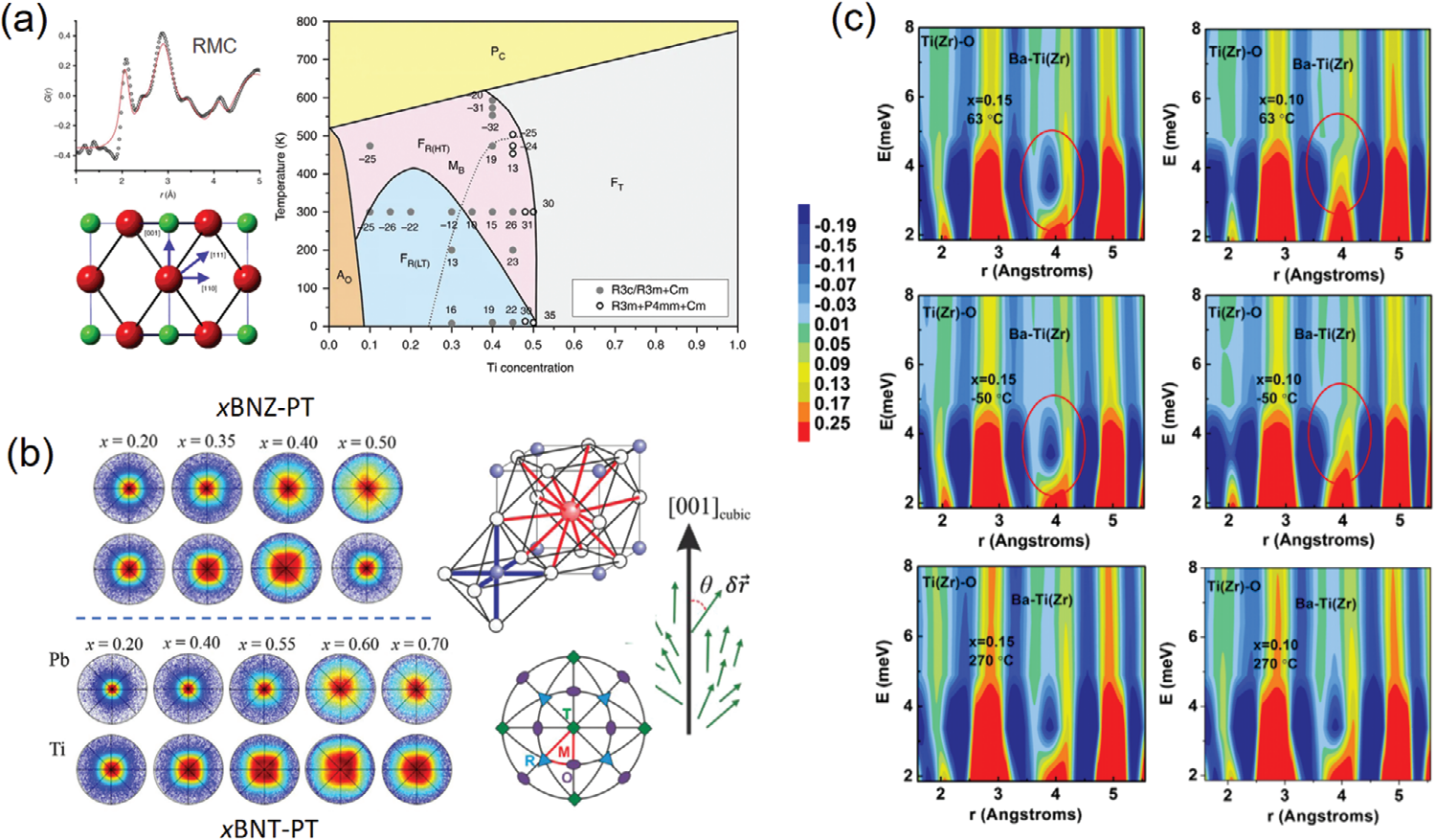
a) The phase diagram of PZT speculated from PDF‐RMC modeling. The crossover between the two intermediate monoclinic phases is marked by a dashed line. Reproduced with permission.^[^ ^150^ ^]^ Copyright 2014, Springer Nature. b) The stereographs of the direction of polar displacements extracted from the RMC simulation of neutron PDF data. The polar direction associated with the perovskite structure is also presented. Reproduced with permission.^[^ ^152^ ^]^ Copyright 2017 by the American Physics Society. c) The contour plots of in situ PDF patterns for different compositions and temperatures. Reproduced with permission.^[^ ^168^ ^]^ Copyright 2018 by the American Physics Society.

The classic family of ferroelectrics is the perovskite‐type (ABO_3_) oxides, such as PbTiO_3_ (PT), BaTiO_3_ (BT), Pb(Mg_1/3_Nb_2/3_)O_3_, BiFeO_3_, (K,Na)NbO_3_, etc., and their composites.^[^ ^148^ ^]^ They usually exhibit an enhanced piezoelectric response at the morphotropic phase boundary (MPB)—a region separating two phases in the compositional phase diagram, where the structure abruptly changes and the electromechanical coupling is maximized.^[^ ^149^ ^]^ Consequently, the structures of piezoelectric materials are practically complex, heterogeneous and very difficult to be clarified, for which the PDF method is frequently used to probe the structures near the piezoelectric MPB. For example, as one typical piezoelectric material, PbZr_1‐x_Ti_x_O_3_ (PZT) was conventionally regarded to show a tetragonal‐to‐rhombohedral transition across the MPB. However, this phase transition is forbidden by the symmetric continuity, and is considered to be mediated with a monoclinic phase. To clarify this intermediate monoclinic structure, neutron PDF coupled with RMC modeling was carried out to study both short‐ and long‐range structures at MPB.^[^ ^150^ ^]^ It is revealed that there are two types of monoclinic phase coexisting in both long‐ and short‐range structures and being separated by an additional phase boundary, which is inspiring for the wide piezoelectric applications (**Figure** **7**a). In addition, the PDF analysis could be also utilized to map the microscopic polarizations (i.e., cation off‐center displacements) in the ferroelectric structure of MPB. By means of the RMC modeling of neutron total scattering, comprehensive pictures of the cation off‐center shifts in a series of ferroelectric compounds could be depicted with varying compositions (Figure 7b).^[^ ^151^, ^152^, ^153^ ^]^ These results provide visual correlations of the local atomic displacements as well as the dynamical coupling between *A*‐ and *B*‐site cations, which promote the uncover of structure‐property relationships in ferroelectrics near MPB.

Relaxor ferroelectrics are a special class of ferroelectrics with disordered structure and peculiar properties.^[^ ^154^ ^]^ One distinguishable feature of the relaxor ferroelectrics is the existence of polar nanoregions (PNRs), that is, nanoscale clusters with randomly orientated dipole moments (polarizations), which affect the local structures and dielectric properties significantly.^[^ ^155^ ^]^ Due to the obvious structural inhomogeneity, clarifying the roles of PNRs played in properties of relaxor ferroelectrics has always been a challenging task. By employing neutron PDF analysis, Jeong et al. reported the temperature evolutions of local polarizations and PNRs in a classic relaxor ferroelectric Pb(Mg_1/3_Nb_2/3_)O_3_ (PMN).^[^ ^156^ ^]^ The PDF results show that PNRs exhibit a medium‐range (≈5–50 Å) rhombohedral order, and the temperature‐dependent volume fraction of the PNRs has been also estimated. Especially, the PNRs were observed to freeze into a spin‐glass state at low temperature (*T* < ≈200 K), which couples with other PDF‐derived local structure evidence to explain the relaxor behaviors. Analogously, the structure of Pb(Zn_1/3_Nb_2/3_)O_3_ (PZN) can be described with a mixture of rhombohedral PNRs in a medium‐range order, as revealed by the PDF‐RMC simulations.^[^ ^157^ ^]^ Yet, the PNRs in PZN own a larger size (≈150 Å) compared to those in PMN, which explains the local correlations and the relaxor behaviors of PZN well. In addition, the short‐range structure correlations, as well as their temperature dependence, in relaxor ferroelectric Sr*_x_*Ba_1‐_ *_x_*Nb_2_O_6_ (SBN) were investigated by neutron PDF analysis.^[^ ^58^ ^]^ It is revealed that the heavily distorted structure is induced by Sr and Ba vacancies, which introduce local strains to tilt the Nb‐O_6_ octahedra and thereby affect the polarizations and relaxor behavior in the material. Apart from the above, the PDF method has been also applied to study the local structural correlations and PNRs in a variety of relaxor ferroelectrics such as BaZr*_x_*Ti_1‐_ *_x_*O_3_,^[^ ^158^, ^159^ ^]^ 0.75PMN‐0.25PT,^[^ ^160^ ^]^ Bi_0.5_K_0.5_TiO_3_,^[^ ^161^ ^]^ and so on.

Nano‐ferroelectrics, such as nano‐BaTiO_3_, are of fundamental interests, as they often show different crystallographic phases and cation off‐center behaviors, as well as unique ferroelectric property compared to the bulk materials. The origin of these features comes mainly from the finite‐size effects that reduce the lattice coherence of the nanoscale systems.^[^ ^162^ ^]^ As a result, the PDF method has been frequently used to study the ferroelectric nanostructures and the related ferroelectric properties. Taking the nano‐BaTiO_3_ as an example, the room‐temperature structure of BaTiO_3_ nanoparticles was proven to be different from the bulk BaTiO_3_ by neutron PDF analysis.^[^ ^163^ ^]^ It is suggested that the average lattice of nanosized BaTiO_3_ appears to be metrically cubic at room temperature, while locally the Ti atoms exhibit a tetragonal‐like off‐center displacement. Additionally, the size‐dependent trends of this Ti off‐center displacement and the related local distortions have been built by X‐ray PDF analysis, indicating that the loss of long‐range coherence is responsible for the cubic symmetry in nanosized BaTiO_3_.^[^ ^164^ ^]^ Afterwards, Rabuffetti et al. examined the local structural evolution of sub‐10 nm BaTiO_3_ nanocrystals upon the synthetic sol‐gel process by X‐ray PDF analysis.^[^ ^165^ ^]^ The results confirm the long‐range cubic symmetry and the local off‐centering of Ti atoms in nanosized BaTiO_3_. Also, the polarization in nano‐BaTiO_3_ is demonstrated to be comparable to that in bulk BaTiO_3_, but the coherent length of the local Ti off‐centering displacement is only ≈16 Å. This suggests that the polarization performance could be achieved in nano‐BaTiO_3_ if the ferroelectric coupling could be increased. Lately, the structures of BaTi_1‐_ *_x_*Fe*_x_*O_3_ (*x* = 0, 0.1, 0.2, 0.3) nanocrystals were systematically studied by X‐ray PDF analysis, and it was revealed that the *c*‐axis of tetragonal *P*4*mm* lattice expands with *x* increasing.^[^ ^166^ ^]^ All these results illustrate that it is of particular importance to consider diffuse signals to elucidate real ferroelectric nanostructures, where the PDF method becomes necessary in many cases.

In recent years, in situ electric‐field PDF technique has been developed to provide insights into the dynamical correlations of atomic structure in novel ferroelectric materials. For example, lead‐free piezoceramics have received much attentions for replacing PZT because they are environmental friendly.^[^ ^142,167^ ^]^ Benefitting from in situ neutron PDF analysis, the stabilization mechanism of PNRs in one of the most general lead‐free piezoceramics, Ba(Zr*_x_*Ti_1‐_ *_x_*)O_3_ (BZT), was clearly revealed (Figure 7c).^[^ ^168^ ^]^ With a proper stoichiometry, the rotation of the local polarization could be sufficiently retarded, hence enabling an enhanced dipole correlation within the PNRs. In another example, the short‐range correlations in (1‐*x*)Ba(Zr_0.2_Ti_0.8_)O_3_‐*x*(Ba_0.7_Ca_0.3_)TiO_3_ (BZT‐*x*BCT), a lead‐free relaxor ferroelectrics, were clarified with in situ X‐ray PDF studies.^[^ ^169^ ^]^ The PDF analysis indicates nonlinear lattice strains over all the compositions, which deviates from the typical piezoelectric behavior. Moreover, the in situ PDF technique has also been employed to investigate the field‐induced structural responses in some other representative ferroelectrics, such as SrTiO_3_,^[^ ^170^ ^]^ (K_x_Na_1‐x_)_0.5_Bi_0.5_TiO_3_,^[^ ^170^, ^171^ ^]^ 0.7PMN‐0.3PT,^[^ ^172^ ^]^ and so on.

#### 3.1.2 Thermoelectrics

Thermoelectric materials are solid‐state energy convertors that enable a direct and reversible conversion between heat and electricity.^[^ ^173^ ^]^ These materials are of great potential to be used for waste heat recovery, refrigeration, and as greenhouse‐gas eliminator, hence providing a feasible pathway to the sustainable energy solution.^[^ ^174^, ^175^, ^176^ ^]^ The efficiency of a thermoelectric material depends on its dimensionless figure of merit (*zT*), defined as:^[^ ^177^ ^]^

(11) $$zT=\frac{S^{2}\sigma }{\kappa }T$$

where *S* stands for the Seebeck coefficient (or thermopower), *σ* is the electrical conductivity, *κ* is the thermal conductivity, and *T* is the absolute temperature. Accordingly, the focus of this research field is to optimize the thermoelectric materials for a large Seebeck coefficient, a high electrical conductivity, and a low thermal conductivity, which are conflicting properties in bulk crystals.^[^ ^178^ ^]^ On the contrary, nanostructured or heterostructured materials with complexity in multi‐scale levels are theoretically predicted to exhibit high *zT*s without compromise.^[^ ^179^ ^]^ For example, the nanoscale materials with confined dimensionality (e.g., nanocrystals, nanowires, films, and so on) are demonstrated to possess an increased Seebeck coefficient and meanwhile a reduced thermal conductivity.^[^ ^180^, ^181^ ^]^ On the other hand, the materials with defects, disorders or heterostructures allow for decoupling of the electrical conductivity with Seebeck coefficient, hence leading to a high *zT*.^[^ ^182^ ^]^ As a result, the high‐efficient thermoelectric materials are usually complex in structures, synthesized in nanoscale, or engineered with heterostructures, defects or local disorders,^[^ ^183^ ^]^ while PDF analysis is often needed to clarify these complex structures that correlate the thermoelectric performance.

The strategy of defect (intrinsic defects or extrinsic dopants) engineering is a very common approach toward high‐*zT* thermoelectric materials. The defects could modify the band structure of a semiconductor for an optimized carrier concentration, which hence enhances the electrical conductivity and also facilitates the scattering of phonons for a suppressed thermal conductivity *κ*.^[^ ^184^ ^]^ For example, the effects of strains on defect‐enabled phonon scattering were examined in thermoelectric SnSe and its alloys.^[^ ^185^ ^]^ By means of PDF analysis, the bonding environments of different SnSe‐based alloys were clearly determined, indicating that the strain fields could make a strong impact on the local disorders and also the thermal conductivity. Similarly, the local structure of thermoelectric Cu_2/3_CrS_2_ possessing 1/3 Cu^+^ vacancies was investigated by X‐ray PDF analysis.^[^ ^186^ ^]^ It was demonstrated that a monoclinic phase with modulated CrS_6_‐octahedral distortions was newly formed, which would impact the *zT* value.

For doped systems, in particular, the Eu^2+^‐substituted defective BaTiO_3_ (i.e., Ba_1‐_ *_x_*Eu*_x_*TiO_3‐_ *_*δ*_*) nano‐powders were proven to show an enhanced thermoelectric performance. With the help of small‐box PDF refinements, the local structures of these substituted materials were found to adopt a disordered *Amm*2 phase within *r* < 20 Å range, while the long‐range structure is identical to the cubic perovskite BaTiO_3_.^[^ ^187^ ^]^ This unique local structure, induced by Eu^2+^ substitution and oxygen defects, enables a remarkably enhanced electrical conductivity of the material, although the parameters of *S* and *σ* go slightly against the overall performance. In another case, the impacts of doping‐derived distortions on the promoted phonon scattering and suppressed thermal conductivity were investigated in Sn_1‐_ *_x_*Ge*_x_*Te series.^[^ ^188^ ^]^ The X‐ray PDF analysis shows that the global cubic structure of Sn_1‐_ *_x_*Ge*_x_*Te presents a local rhombohedral distortion accompanied with Ge alloying, which is fingerprinted by a local chain‐like Ge off‐centering. This local structural change could effectively scatter acoustic phonons, giving rise to a low *κ* and an improved thermoelectric performance. Besides, the structural complexity in Yb_21_Mn_4_Sb_18_ thermoelectric system was probed by neutron PDF measurements.^[^ ^189^ ^]^ The small‐box refinement results indicated a distorted structure with anisotropic atomic displacements, which is consistent with a new Zintl phase that is first discovered in the Yb‐Mn‐Sb system.

The anharmonicity of interatomic potential well, measured by thermal expansion property macroscopically and Grüneisen parameter *γ* microscopically,^[^ ^148^ ^]^ is a fundamental to suppress the thermal conductivity. This is realized by limiting the phonon lifetime for a reduced *κ*.^[^ ^190^ ^]^ A strong anharmonicity could be achieved from weak or heterogeneous bonding environments, as well as heavy or lone‐pair atoms. For example, the *n*‐type rock‐salt AgPbBiSe_3_ was found to exhibit a low *κ*. The X‐ray PDF results reveal an obvious bonding heterogeneity existing in the lattice, which could be originated from the lone‐pair 6*s* ^2^ electrons of Pb and Bi atoms.^[^ ^191^ ^]^ This induces strong anharmonicity that greatly enhances the phonon‐phonon scattering, which is responsible for the reduced thermal conductivity. In another *n*‐type thermoelectric AgBiS_2_, the *κ* value could almost approach to it theoretical minimum. Analogously, the lone‐pair electrons of Bi atoms were found to play a critical role to this phenomenon, which leads to local structural distortions along the [011] direction and thereby a strong lattice anharmonicity, as revealed by the X‐ray PDF analysis.^[^ ^192^ ^]^

As mentioned, high *zT* values are expected in nanoscale or low‐dimensional thermoelectric materials. The underlaid mechanism is the quantum size effect that could reduce the mean free path of phonon scattering.^[^ ^193^ ^]^ The PDF method is frequently used to probe the nanostructures and clarify the related fundamentals. For example, Soriano et al. combined thermoelectric PbTe and Sb_2_Te_3_ into solid solutions (i.e., Pb*_m_*Sb_2_ *_n_*Te*_m_*_+3_ *_n_*), and found that these compounds are stable only when they are nanocrystals.^[^ ^194^ ^]^ The PDF analysis suggests that the local structure of Pb*_m_*Sb_2_ *_n_*Te*_m_*_+3_ *_n_* is distorted relative to the rock‐salt cubic phase, which provides key clues for understanding the thermodynamic limits of nanoscale systems. In a separate study focusing on thermoelectric Li_1‐_ *_x_*Sn_2+_ *_x_*As_2_, the PDF analysis was carried out to probe the phase segregation at a nanoscale level.^[^ ^195^ ^]^ It is indicated that the low thermal conductivity in Li_1‐_ *_x_*Sn_2+_ *_x_*As_2_ could be attributed to the nano‐inclusions and abundant boundaries associated with Sn_4_As_3_ and SnAs nanodomains, resulting in a hierarchical superstructure that scatters the heat‐carry phonons. In addition, similar nanophase segregation was also found in PbTe_1‐_ *_x_*S*_x_* compounds.^[^ ^196^ ^]^ The small‐box Rietveld PDF refinements show that the system includes nanoscale PbTe‐rich and PbTe‐poor regions, which plays an important role in producing enhanced *zT* in this material.

In some cases, the thermoelectric materials may present amorphous state at the beginning or during the thermal processes, where PDF method is essential for studying these materials. For example, carbonized polydopamine (cPDA) was found to have potential for thermoelectric applications, so clarifying the atomic structure and its thermal evolutions of cPDA are highly desirable. By fitting the neutron PDF patterns with a graphite structure model, the short‐range atom correlations were characterized at the small *r*‐distance region.^[^ ^197^ ^]^ Although the structure of cPDA is mainly amorphous, a short‐range ordering was revealed within a nanoscale region of ≈0.6 nm. Also, a graphite‐like local structure was observed to emerge in cPDA by increasing the temperature of heating treatment. As an opposite example, the X‐ray PDF was measured in thermoelectric Mg_3_Sb_1.475_Bi_0.475_Te_0.05_ to exclude the possible amorphous‐Bi formation during the heat cycling.^[^ ^198^ ^]^ It is revealed that a crystalline Bi impurity, instead of Bi amorphous phase, appears upon cooling from 725 K, which is conducive to paint a clear picture of the thermal stability in this material.

**Figure 8:**
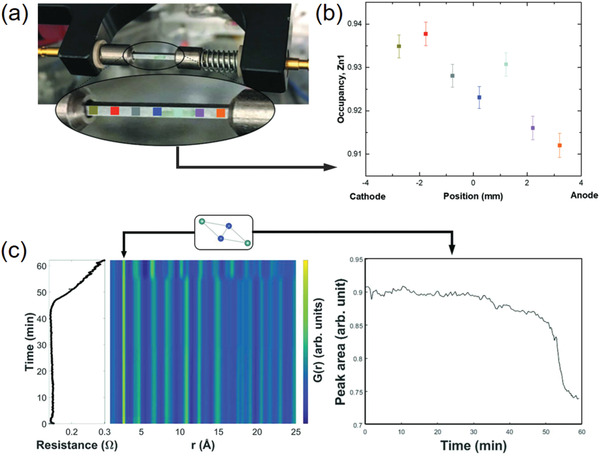
a) The photo of experimental setup for in situ PDF measurements, installed at P02.1 beamline, DESY. The sample is loaded on the pathway of X‐ray beam between two electrodes. b) The refined Zn occupancy at different positions colored in (a). c) The contour plot of the collected in situ PDF patterns along with the change of electrical resistance. The integration of the PDF at ≈2.7 Å, associated with the Zn occupancy, is also performed. Reproduced under the terms of CC‐BY Creative Commons Attribution 4.0 International License.^[^ ^199^ ^]^ Copyright 2020, The Authors. Published by International Union of Crystallography.

Very recently, inspiring progress has been made in developing synchrotron‐based in situ PDF technique dedicated for studying thermoelectric materials. **Figure** **8** shows the in situ setup that is capable of collecting X‐ray PDF data with real‐time detection of electrical resistance on pallets subjected to electrical current.^[^ ^199^ ^]^ With this method, the in situ PDF investigation of *β*‐Zn_4_Sb_3_ thermoelectric material was carried out at different current densities, and both zinc‐ion migrations and related decomposition reaction were evaluated. Based on its principles, this in situ PDF setup could also be potentially used for studying ferro‐/piezo‐electric materials and solid‐state battery electrolytes.

### 3.2 Colossal Magnetoresistive Manganites

**Figure 9:**
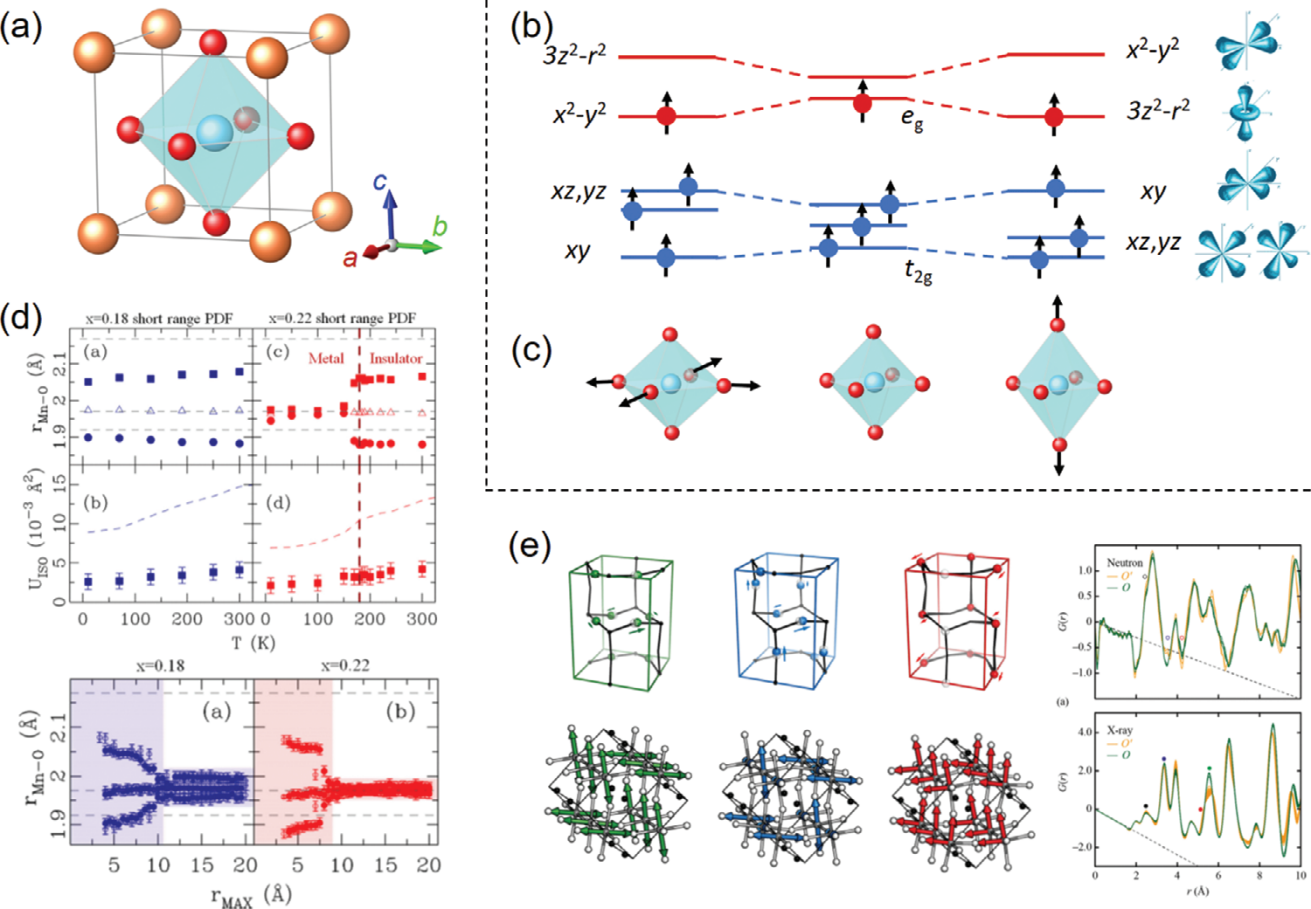
a) The near‐cubic unit cell of the *R*_1‐_ *_x_A_x_*MnO_3_ perovskite. b) The schematic diagram of the five‐fold degenerated Mn^3+^‐3*d* orbital levels with distinct crystal‐field splittings. c) The typical octahedral Jahn–Teller distortions coupled with *e*_g_ occupied orbitals. d) The results of neutron PDF refinements for La_1‐_ *_x_*Ca*_x_*MnO_3_ (*x* = 0.18 and 0.22) samples at variable *r*‐distances to reveal the correlation length of the J–T distortion. Reproduced with permission.^[^ ^212^ ^]^ Copyright 2016 by the American Physics Society. e) The representative orbital‐correlated models for small‐box PDF refinements and the corresponding X‐ray and neutron PDF patterns. Reproduced with permission.^[^ ^213^ ^]^ Copyright 2017 by the American Physics Society.

The term of CMR refers to a large drop of resistance in a magnetic material driven by application of a magnetic field near the Curie temperature (*T*_C_), in which the material would undergo a M–I transition accompanied with the intrinsic ferromagnetic‐to‐paramagnetic (F–P) transition.^[^ ^200^, ^201^, ^202^ ^]^ The growth of research interest in this area comes from the prospective usage of disk read‐and‐write heads, whose performance is expected to exceed that of the giant magnetoresistance (GMR) devices that are composed of transition‐metal (TM) multilayer hybrids.^[^ ^203^ ^]^ Mostly, the CMR effect is observed in mixed‐valent manganite (Mn^3+^‐Mn^4+^) perovskite, *R*_1‐_ *_x_A_x_*MnO_3_, where *R* is a trivalent lanthanide metal (e.g., La, Pr, Nd) and *A* is a divalent cation (e.g., Ca, Sr, Ba, Pb). In these materials, the mixed Mn^3+^‐Mn^4+^ ions occupy the central sites of the vertex‐sharing MnO_6_ octahedra in the near‐cubic units (**Figure** **9**a). It is now well established that at the heart of the CMR physics lies a strong electron‐phonon coupling mediated via a J–T distortion of the Mn^3+^ ions. On account of the crystal field, the five‐fold degeneracy of 3*d* orbitals is partially lifted into three lower‐lying *t*_2g_ levels and two higher‐lying *e*_g_ levels. For the Mn^3+^O_6_ octahedra, the central Mn sites present a high‐spin $t_{2g}^{3↑}e_{g}^{1↑}$ electronic configuration, wherein the occupied *e*_g_ level shows a marked tendency to lower its energy by octahedral distortion (Figure 9b).^[^ ^204^ ^]^ This J–T distortion is regarded as the key enabler to localize the *e*_g_ charge carriers when *T > T*_C_, and competitively these carriers could be released by the ferromagnetic exchange interactions of Mn spins at *T*_C_.^[^ ^205^ ^]^ Such a competition between the localization and delocalization of charge barriers drives the coincident M–I transition, whose balance induces complex local structures in the CMR manganites. Apart from the CMR effect, the cooperative J–T distortion also leads to some unique phenomena such as orbital and charge ordering,^[^ ^206^ ^]^ phase segregations,^[^ ^207^ ^]^ etc., introducing additional lattice complexity in the materials.

The most general octahedral J–T distortions are the elongation along *z*‐axis coupled with 3*z* ^2^‐*r* ^2^ orbital or along *x‐* and *y*‐axis (in‐plane) coupled with *x* ^2^–*y* ^2^ orbital (Figure 9c).^[^ ^208^ ^]^ A quantitative probe of these distortions is a key step toward understanding the related CMR physics. With this regard, the PDF method enables a straightforward way to distinguish the J–T distortion with the first Mn‐O peak being split into two components. This has triggered a lot of PDF investigations on the CMR manganites with temperature and doping leveling out across the M–I transition boundary.^[^ ^209^, ^210^, ^211^ ^]^ For example, Shatnawi et al. conducted a comprehensive study on the local J–T evolutions in the La_1‐_ *_x_*Ca*_x_*MnO_3_ (*x* = 0.18 and 0.22) compounds across their M–I transition.^[^ ^212^ ^]^ A two‐phase structural model was applied to describe the neutron PDF patterns, and the refinements were carried out at variable *r*‐distances for revealing the correlation length of the J–T distortion (Figure 9d). The results show that the J–T distortion suddenly vanishes as soon as the temperature or doping level goes into the metallic phase region. Additionally, the two‐phase modeling provides no evidence of a phase coexistence away from *T*_C_, which disagrees the previously assumed percolative scenario for the M–I transition based on nanoscale phase separation. Recently, Thygesen et al. tracked the orbital order/disorder transition that couples the J–T distortions in LaMnO_3_, implemented by combining X‐ray and neutron PDF analysis at variable temperatures.^[^ ^213^ ^]^ Different orbital‐correlated models were tested for small‐box PDF refinements, and a three‐state Potts (3SP) model was revealed to describe the *G*(*r*) patterns best (Figure 9e). The results show that a discontinuous local structural change emerges between the ordered and disordered states, which differs from the conventional order/disorder descriptions.

On the other hand, the lattice polarons, which is formed in response of the M–I transition by an isolated *e*_g_ charge localizing on Mn^3+^, are believed to play a key role in the CMR effect.^[^ ^214^ ^]^ These polarons are distributed randomly within short‐range orbital and charge correlations (<20 Å), leading to heterogeneous local structures along with the J–T distortion. Clearly, a direct observation of the polaronic state is difficult but necessary for the accessible CMR mechanism. In this respect, Billinge et al. performed a series of neutron PDF analysis on La_1‐_ *_x_*Ca*_x_*MnO_3_ to reveal the local structural changes within the temperature range of the M–I transition.^[^ ^215^ ^]^ An abrupt broadening of the nearest Mn‐O and O—O peaks could be observed close to *T*_C_, enabling an indicator of the *e*_g_ charge localization that fingerprints the lattice polarons. For a further step, the PDF patterns are proven to be well modeled with a polaronic structure, offering quantitative oxygen displacements of the polaron distortion (*δ* ≈ 0.12 Å). In addition, the neutron PDF analysis could be also applied to evaluate the polaron stability in different conditions. By fitting the first PDF peak for different manganites (*A*_0.7_ *A′*_0.3_MnO_3_, *A* = Pr, Y, La, *A′* = Ca, Ba, Sr), Louca et al. showed that the polaron stability could be modified by the optimum size of *A*‐site ions.^[^ ^216^ ^]^ In this way, the CMR effects at low temperature are suggested to be adjusted in terms of the local structure.

### 3.3 High‐Temperature Superconductors

High‐temperature superconductors, referred to as high‐*T*_c_ superconductors or abbreviated as HTSCs, are the typically defined as materials that present a superconductor behavior above 77 K.^[^ ^217^ ^]^ This is actually the lowest temperature achievable by liquid‐nitrogen cryostat, enabling its possible applicability in practice. At present, the well‐studied HTSCs are mostly ceramic materials with layered structures, including cuprates, iron‐based compounds, MgB_2_ analogues, and so on. The superconductivity in these strongly correlated systems shows a complex interplay with their structures characterized by spin, orbital, and magnetic orders. In particular, the local structures without long‐range ordering, such as local distortions, boundaries/interfaces, chemical doping, twinning, anionic defects, have a strong impact on the critical *T*_c_ temperature.^[^ ^218^ ^]^ In this context, PDF analysis can play an important role in the fundamental understanding of the complex interplay between spin, structure, and superconductivity, which will surely facilitate both the theoretical and practical approaches for high‐performance HTSCs.

**Figure 10:**
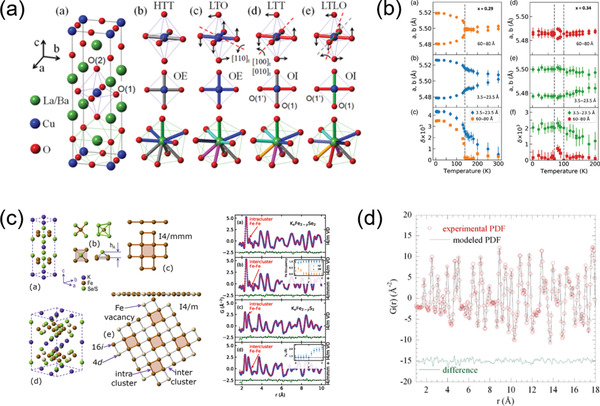
a) Structural details of La_2‐_ *_x_*Ba*_x_*CuO_4_ in high‐temperature tetragonal *I*4/*mmm* (HTT), low‐temperature orthorhombic *Bmab* (LTO), low‐temperature tetragonal (*P*4_2_/*ncm*), and low‐temperature less orthorhombic *Pccn* phases. Reproduced with permission.^[^ ^224^ ^]^ Copyright by the American Physics Society. b) The refined local lattice parameters at variable temperatures and different fitting ranges. Reproduced with permission.^[^ ^231^ ^]^ Copyright by the American Physics Society. c) The structural models of K*_x_*Fe_2‐_ *_y_*Se_2‐_ *_z_*S*_z_* superconductor with local disorder and Fe vacancies and the fits of the structural models to the PDF data. Reproduced with permission.^[^ ^234^ ^]^ Copyright by the American Physics Society. d) The PDF refinements of the MgB_2_ *G*(*r*) pattern collected at 300 K. Reproduced with permission.^[^ ^237^ ^]^ Copyright 2012, Springer Nature.

Cuprate‐based superconductors are the most classic HTSC that dates back to the discovery of La_5‐_ *_x_*Ba*_x_*Cu_5_O_5(3‐_ *_y_*_)_ in the year 1986.^[^ ^219^ ^]^ For these cuprate compounds, their superconducting properties are governed by the quasi‐2D CuO_2_ plains, in which the electrons are transferred via weakly coupled Cu—O bonds. As a result, the bonding geometry of the CuO_6_ octahedron impacts the superconductivity of cuprates greatly, as it couples the band structure and energy gap of the materials. Especially, the oxygen non‐stoichiometry that lies in the heart concept of the cuprate‐based superconductors.^[^ ^220^ ^]^ The oxygen defects in the lattice are capable of shaping a local octahedral structure and therefore the superconductivity. For example, YBa_2_Cu_3_O_7‐_ *_y_* is an insulator with a tetragonal structure for 0.65 < *y* < 1. When the *y* value reaches 0.65, the Cu—O chains are linked with occupied O(1) sites, and the material becomes superconductive.^[^ ^221^ ^]^ Given the strong response of a band structure to the Cu—O bonding geometry, a precise determination of the local structure in terms of CuO_6_ octahedra enables a bridge between non‐stoichiometric composition and superconductivity, for which the PDF technique can play an important role for investigation. By means of a neutron PDF analysis, Božin et al. studied the tilts of local CuO_6_ octahedra in a typical HTSC system La_2‐_ *_x_*Sr*_x_*CuO_4_ (0 ≤ x ≤ 0.3).^[^ ^222^ ^]^ The results show that the degree of local octahedral tilts decreases continuously as a function of Sr content, which provides evidence of the striped charge distribution in short‐range orders. A further PDF investigation reveals that the doping Sr into the lattice of La_2‐_ *_x_*Sr*_x_*CuO_4_ will trigger a broadening of in‐plain Cu—O bond distribution.^[^ ^223^ ^]^ A charge inhomogeneous state is therefore supported by the PDF results, building a bridge between the doping effect and superconductivity in this material. Furthermore, there exists a long‐term puzzle about the contradiction of local‐ and long‐range CuO_6_ octahedral tilt correlation in doped cuprate superconductors (**Figure** **10**a). This fundamental issue has been addressed by combining neutron PDF with a neutron diffraction technique as function temperature.^[^ ^224^ ^]^ For the La_2‐_ *_x_*Ba*_x_*CuO_4_ compound, these two approaches offer distinct evidence of the tetragonal tilts in low‐temperature orthorhombic phase, as well as the local tilt fluctuations in the high‐temperature tetragonal phase. This has provided as an important guidance of the electron‐phonon coupling in optimizing this strong‐correlation superconducting system.

As a new type of HTSC system, the iron‐based superconductors have attracted more and more attentions since first discovered in 2006.^[^ ^225^ ^]^ Later in 2008, iron‐pnictogen compound, that is, LaFeAsO, were found to show superconductivity,^[^ ^226^, ^227^ ^]^ which is currently prominent in the family of iron‐based HTSC. Similar with the cuprate compounds, the superconductivity of the iron‐based HTSC relies on the metallic edge‐sharing FeAs_4_ tetragonal layers. It has been well acknowledged that superconducting properties in this material become unstable when the anti‐ferromagnetic (AF) order is disrupted to the paramagnetic (PM) phase.^[^ ^228^ ^]^ In this magnetic transition, the AF order is coupled with an orthorhombic lattice disorder, while the PM structure is tetragonal.^[^ ^218^ ^]^ This combined structural and magnetic transition has long been a central topic in these iron‐based materials.^[^ ^229^, ^230^ ^]^ In this respect, the PDF technique has been demonstrated as a powerful tool to elucidate the interplay between structure, magnetism, and superconductivity. For example, by virtue of temperature‐dependent PDF measurements, the local structure of Sr_1‐_ *_x_*Na*_x_*Fe_2_As_2_ was found to differ from the average tetragonal symmetry, consisting of a short‐range orthorhombic region with a length scale of 2 nm (Figure 10b).^[^ ^231^ ^]^ This has explained the high orthorhombic stability and its correlation with the superconducting property in this material. In another case, the local structure of two similar iron‐based superconductors, Ca_0.82_La_0.18_FeAs_2_ (referred as 112) and Ba_0.64_K_0.36_Fe_2_As_2_ (referred as 122) were investigated by X‐ray PDF analysis.^[^ ^232^ ^]^ It is shown that the Fe‐As conductive layers in both systems is similar to typical iron‐based materials, but they exhibit a local deviation from the perfect structural symmetry. These results suggest that the keys toward modulating the superconducting properties of iron‐based HTSC lay in the nanoscopic region. Li et al. combined the EXAFS and PDF techniques to study the Fe‐As local structure in BaFe_2_As_2_ and LiFeAs systems.^[^ ^233^ ^]^ They found that the local distortion in the FeAs planes of BaFe_2_As_2_ exhibits a temperature‐dependent feature upon decreasing to *T*_C_, which supports the local orthorhombic structure (*I*2*mm*) observed previously. In the case of LiFeAs, no structural transition occurs with the superconducting transition (≈18 K), and only a slight distortion could be observed below 200 K. These results further indicate the importance of FeAs distortion to the superconductivity of iron‐based HTSCs.

In addition, the compositional non‐stoichiometry, as mentioned above, plays a critical role in the superconducting properties. By neutron PDF analysis, Mangelis et al. recently studied the local structure and disorders of K*_x_*Fe_2‐_ *_y_*Se_2‐_ *_z_*S*_z_*, and found that the interplay of magnetic and superconductivity relies on the Fe‐vacancy ordering (Figure 10c).^[^ ^234^ ^]^ By modeling the PDF data with small‐box refinement and a large‐box simulation, a high‐degree of structural complexity was revealed on a nanoscopic length scale. It is suggested that the substantial Fe‐vacancies can modify the bond length distribution. These results have offered a deeper understanding of coupling between Fe‐vacancy ordering and the superconducting properties in this iron‐based material.

Magnesium diboride (MgB_2_), as another important type of HTSC material, is a multiband superconductor with a *T*_c_ of ≈40 K first discovered in 2001. The structure of MgB_2_ is staggered with Mg triangular stacking and boron honeycomb hexagonal planes, which couples a two‐gap superconducting model within the boron layers.^[^ ^235^ ^]^ It is now realized that the HTSC property of MgB_2_ is highly relevant with the local structure of boron sublattice, but the experimental evidence on this point is still lacking. Although the average structure of MgB_2_ (*P*6/*mmm*) could be revealed by XRD, the local structure especially the fluctuations of B‐coordination has not been clearly clarified. Due to the poor X‐ray scattering capability of B‐element, the neutron PDF plays an irreplaceable role in studying the local structure of MgB_2_. For instance, the long‐ and short‐range structures of MgB_2_ and Mg_0.5_Al_0.5_B_2_ were investigated with high‐resolution neutron PDF.^[^ ^236^ ^]^ It is suggested that the impacts of Al dopant on local structure and *T*_c_ are twofold: i) the filling of electronic states in the 2D *σ*‐band of *p_xy_*‐orbitals within the boron layers, and ii) the high‐degree of lattice disorder for anomalous short‐range lattice dynamics. In another case, the neutron PDF investigation on the Mg_1‐_ *_x_*Al*_x_*B_2_ revealed a phase separation in this system, which can be described by an inhomogeneous structure with local distortions (Figure 10d).^[^ ^237^ ^]^

### 3.4 Quantum Dots

The concept of QDs, as a central topic of nanotechnology, refers to fragments of semiconductor containing hundreds to thousands of atoms. This type of nanoscale material usually possesses unique optical and electronic properties due to quantum mechanics, enabling them to be potentially used in the fields of light‐emitting diodes, solar cells, photocatalysts, and so on.^[^ ^238^, ^239^, ^240^ ^]^ The optoelectronic characteristics of QDs are size‐dependent—the electrons or electron holes are constrained within the dimension of QDs, which enlarges the band gap for tunable electrical and optical properties. With this regard, the QD lattice is short‐range ordered without long‐range coherence, so the diffraction‐based crystallography fails in determining the QD structures. On the other hand, the nanoscale QDs possess bulk‐like bonding geometry and a relaxed surface reconstruction with defects, which brings additional challenges for structural characterization. The PDF extracted from X‐ray and neutron total scatterings has been proven effective for encoding short‐range structures. In the field of QDs, the PDF technique is typically used to probe inhomogeneous bonding environment, phase segregation, structural defects, etc., which are induced by a finite‐size effect and highly correlated to the optoelectronic properties.

**Figure 11:**
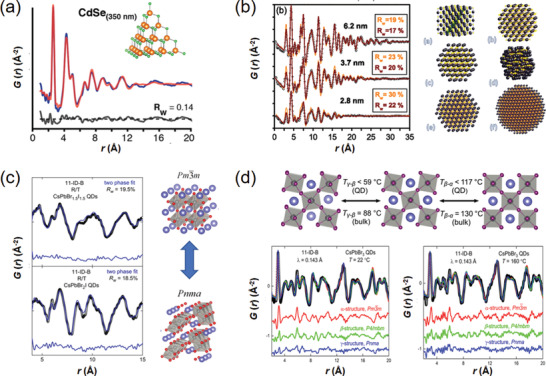
a) The PDF refinement pattern of CdSe QDs. The inset is the confined zinc‐blende pyramidal model adopted in the refinement. Reproduced with permission.^[^ ^242^ ^]^ Copyright 2014, American Chemical Society. b) The PDF refinement patterns of PbSe QDs based on both fully stoichiometric (orange line) and off‐stoichiometric models. The confined models with different surface terminations are also presented. Reproduced with permission.^[^ ^243^ ^]^ Copyright 2010 by the American Physics Society. c) The PDF refinement patterns of CsPbBr_1.5_I_1.5_ and CsPbBr_2_I QDs based on two‐phase model containing *α*‐phase (*Pm*‐3*m*) and *δ*‐phase (*Pnma*). Reproduced with permission.^[^ ^250^ ^]^ Copyright 2016, American Chemical Society. d) The schematic diagram of temperature‐dependent phase transitions for bulk and QDs, along with the PDF refinement patterns with different structural models at 22 and 160 °C. Reproduced with permission.^[^ ^251^ ^]^ Copyright 2018, American Chemical Society.

Gaining insight into the local disorders in QDs is an important aspect of PDF research field, since these disorders enable direct modification of band structures. For example, the local disorders and crystallinity of 3.4 nm ZnS nanoparticles were quantitatively determined by PDF analysis.^[^ ^241^ ^]^ By comparing the experimental *G*(*r*) profile with an ideal bulk pattern truncated at *r* = 3.4 nm, the atomic displacements could be extracted by excluding the contribution of thermal displacements. These inhomogeneous lattice strains, which may be an indicator of an unsatisfied coordination on the surface, could stiffen the Zn—S bonds and modify the electron structure. In addition, the structural inhomogeneity in the CdSe clusters were also studied by PDF analysis combined with singe‐crystal XRD.^[^ ^242^ ^]^ The results reveal that the cores of clusters are built by {111}‐terminated pyramidal nanostructures with a zinc‐blende packing of atoms (**Figure** **11**a). In another case, the PDF analysis was employed to study the local disorders in PbSe nanocrystals as a function of particle sizes.^[^ ^243^ ^]^ By refining the *G*(*r*) patterns with various finite‐size models, an Se‐deficient model reproduces the experimental PDF data best. This means that the local structure of nanosized PbSe is distorted and off‐stoichiometric, which cannot be simply regarded as the conventional rock‐salt coherence (Figure 11b). Similarly, for the nanoscale Pb*_m_*Sb_2_ *_n_*Te*_m_*_+3_ *_n_* system, the cubic rock‐salt PbTe model also failed to describe the short‐range *G*(*r*) profile (*r* < 7 Å), indicating a local structural distortion within the short‐range ordering.^[^ ^195^ ^]^

In addition, the local disorders in QDs are usually described by phase changes deviated from the bulk, which could be distinguished by PDF analysis. As a class of important QDs, nanocrystal cadmium chalcogenides, such as CdS and CdSe and CdTe, are typical examples with distinct structures against the bulk. It was found that the PDF pattern of CdSe nanoparticles cannot be fitted well with the bulk models of either hexagonal wurtzite or cubic zinc blende structures.^[^ ^244^, ^245^ ^]^ Instead, a two‐phase model combining both the bulk structures enables a better fitting of the PDF data with a reduced *R*_w_ value. The coexistence of two bulk phases could be explained by the random stacking of layers in the CdSe nanostructure. Besides, the QD structures of CdS and CdTe were found to be sensitive to the surface capping agents used in the synthetic process.^[^ ^246^ ^]^ Based on the small‐box PDF refinements, the CdSe nanocrystals that are capped with oleic acid exhibit the zinc blend ordering with only slight distortion, while the thiol‐capped CdTe shows a heavily distorted structure due to the significant difference between surface and bulk. To describe this core‐shell heterogeneity, a mixture of two zinc‐blend models is more appropriate to reproduce the *G*(*r*) pattern of PDF data.

The defect chemistry in QDs is a fundamental but challenging research field. These defects, especially the stacking faults in layered chalcogenides, show the potential to modify the band structure for desirable optoelectronic properties. With the help of real‐space Rietveld PDF refinements, the size‐dependent structure of stacking faults in CdSe nanocrystals could be well determined.^[^ ^247^ ^]^ The results show that the core structure of the CdSe nanoparticles could be described with the hexagonal wurtzite structure containing local disorders and ≈50% stacking faults density. This will induce significant lattice strain that interplays with the band structure. In addition, the stacking faults in the core of ZnS nanoparticles were probed by the PDF coupled with TEM measurements, which draws a clear picture of the atomic structure of this material.^[^ ^248^ ^]^ Furthermore, the chalcogenide QDs are usually grown with a core‐shell architecture consisting of two distinct components, for which the strain and stacking faults could be induced by lattice mismatch. As a model system, the CdSe/CdS core‐shell nanorods were studied by combining high‐angle annular dark‐field (HAADF) STEM and PDF techniques.^[^ ^100^ ^]^ The results show that the lattice strain and stacking fault density are strongly correlated with the size and shape of the CdSe cores. A smaller and spherical core is beneficial to reduce the strain and stacking faults.

On the other hand, halide‐based perovskites have demonstrated their great promise for the usage of high‐efficient photovoltaic devices.^[^ ^249^ ^]^ The structure of halide perovskites depends on their composition and temperature, which can be clearly elucidated by PDF investigations. In this respect, several research works have focused on the local structures of CsPbX_3_ (X = Cl, Br, and I) QDs.^[^ ^248^, ^249^, ^250^, ^251^ ^]^ By means of small‐box PDF refinements, the structure of colloidal CsPbBr*_x_*I_1‐_ *_x_* has been identified as a function of *x*.^[^ ^250^ ^]^ The results indicate that the CsPbBr*_x_*I_1‐_ *_x_* QDs contain both cubic (*Pm*‐3*m*) and orthorhombic (*Pnma*) phases coexisting in the lattice over a wide range of *x* (Figure 11c), offering a deeper understanding of the composition‐dependent photophysics in this material. In addition, the temperature‐dependent phase transitions in CsPbBr_3_ QDs were studied by variable‐temperature PDF analysis.^[^ ^251^ ^]^ Different from the bulk CsPbBr_3_ that exhibits orthorhombic‐to‐tetragonal (*T*_c_ ≈50–59 °C) and tetragonal‐to‐cubic (*T*_c_ ≈108–117 °C) transitions at specific temperature ranges, the CsPbBr_3_ nanocrystals undergo these two transitions at lower temperatures (Figure 11d). From the local structural point of view, an orthorhombic distortion is expected over the temperature range of 22–160 °C, and the degree of distortion changes with temperatures. These results clarify the order–disorder nature of the transitions in the CsPbBr_3_ QDs. On the other hand, the rotation of PbX_6_ octahedra in CsPbX_3_ shows a strong impact on the optoelectronic properties by coupling the electronic structure. By virtue of PDF analysis, the CsPbBr_3_ nanocubes show a slight rotation of corner‐shared octahedral frames compared with the bulk reference.^[^ ^252^ ^]^ In view of this, the nanostructure of CsPbBr_3_ could be identified as orthorhombic (*Pnma*) phase rather than the bulk‐like cubic (*Pm*‐3*m*) phase. Doping zinc into the lattice of CsPbI_3_ QDs improves the photoluminescence quantum yielding (PLQY), but its detailed structure regarding the zinc doping sites is unclear. By Gaussian fitting of the PDF peaks, the Zn^2+^ dopant is revealed to be doped interstitially in the perovskite lattice.^[^ ^253^ ^]^

### 3.5 Nano‐Catalysts

In chemistry, catalysts act as chemical promoters to accelerate reaction processes without being consumed, which can be roughly classified, based on the reactions employed, as electrocatalysts, organocatalysts, photocatalysts, and enzymes. As is well known, modern catalysts are typically synthesized at nanoscale with a high specific surface area, as it enables a greater number of catalytic active sites.^[^ ^254^ ^]^ These nanomaterials are lacking in long‐range ordering, so their catalytic activities depend largely on the short‐range structures with inhomogeneities in elemental, phase, and bonding distributions. With this regard, it is of critical importance to precisely determine the structural inhomogeneity of nano‐catalysts, in order to interplay with the electronic structure for a clear and predictable redox sequence. As mentioned above, the conventional diffraction‐based techniques are incapable of encoding the structures without long‐range periodicity, so the complementary technique of PDF deserves to be undertaken in the research field of catalysts. In this section, a brief introduction regarding the PDF investigations on probing the structures of nano‐catalyst is given as follows.

Noble‐metal electrocatalysts that enable fuel cells and metal‐air batteries are crucial for the technical developments of cleaner energy conversion and manufacturing.^[^ ^255^ ^]^ However, the broad applications of noble‐metal catalysts in current fuel‐cell technology are hindered by the sluggish kinetics and high cost of noble metals.^[^ ^256^ ^]^ As a result, substantial efforts have been devoted to alloying transition metals (TMs) for improved activity and lowered cost, leading to a fascinating family of bimetallic nanoalloys as state‐of‐the‐art electrocatalysts. Despite the partial success, nevertheless, the activity and stability of noble‐metal‐TM nano‐catalysts are still awaited to be substantially optimized for the broader commercial success.

**Figure 12:**
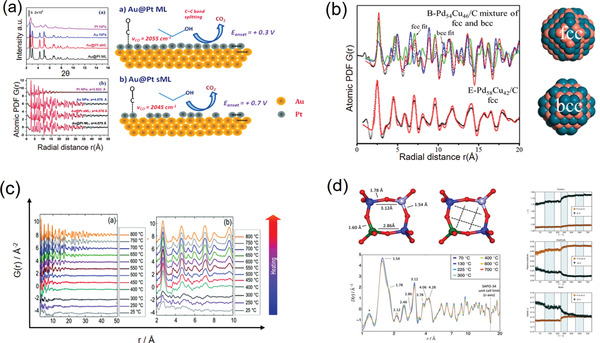
a) The experimental and refined PDF patterns, as well as the schematic diagram of the Pt layers of Au@Pt ML and Au@Pt sML electrocatalysts. Reproduced under the terms of CC‐BY Creative Commons Attribution 3.0 International License.^[^ ^260^ ^]^ Copyright 2014, The Authors. Published by the PCCP Owner Societies. b) The PDF refinements for the data collected from Pd_54_Cu_46_/C and Pd_58_Cu_42_/C nanoparticles. The atomic configurations show the confined structural model of chemically disordered *fcc* (space group: *Fm*‐3*m*) and ordered *bcc* (space group: *Pm*‐3*m)* phases. Reproduced with permission.^[^ ^266^ ^]^ Copyright 2015 by the American Physics Society. c) In situ PDF data of the PtNi precursor collected at variable temperatures elevated from room temperature to 800 °C. Reproduced under the terms of CC‐BY Creative Commons Attribution 3.0 International License.^[^ ^270^ ^]^ Copyright 2018, The Authors. Published by the PCCP Owner Societies. d) The PDF patterns with *r* distance from 0.9 to 20 Å collected at different temperatures. The local bonding geometries from the SAPO‐34 unit cells are also presented. The right column shows the variations in the Al‐O, P‐O (and Si−O) bond lengths (top), as well as their corresponding peaks amplitude (middle) and width (bottom). Reproduced with permission.^[^ ^274^ ^]^ Copyright 2018, Wiley‐VCH.

Local geometric and electronic structures have been proved to show a strong correlation with the activities of TM‐alloyed electrocatalysts. It has been shown that the local structure (i.e., distance and coordination between atoms) would modify the electronic structure by strain effect, where the electronic structures and relative band positions could be altered for promoted adsorbate‐surface interaction.^[^ ^257^, ^258^, ^259^ ^]^ However, accurately determining the local structures (e.g., bond length and coordination numbers) in the nanoscale TM‐alloyed electrocatalysts is a critical challenge, which hinders the catalytic reaction‐oriented design and mechanism studies. The PDF technique has been proven as a powerful tool for studying the strain through probing atomic displacements. For example, the nanoscale electrocatalyst, consisting of a Pt monolayer (ML) and sub‐monolayer (sML) deposited on Au nanoparticles (Au@Pt ML/sML), was studied by X‐ray PDF analysis.^[^ ^260^ ^]^ It was found that the strain effect due to lattice mismatch within Pt‐Au hybrid is closely related to the electrochemical activity. The results show that the lattice of Au@Pt sML match well with that of Au nanoparticles, while the lattice parameters of Au@Pt ML contract compared to those of Au (**Figure** **12**a). Accordingly, the induced lattice strain in Au‐supported Pt ML has a significant impact on the electrocatalytic behavior. In addition, the local structure of PtNi/C catalysts annealed in different atmospheres were investigated by Rietveld refinement of PDF.^[^ ^261^ ^]^ The results show that the thermally annealed PtNi/C nanoparticles maintain the *fcc* structure, but the lattice parameters are significantly relaxed under different gas atmospheres. As a result, the strains differ in different samples, which have an important influence on the activity of oxygen reduction reaction (ORR). Zhong et al. investigated the lattice strains of Pt‐Ni nanowires with composition‐tunable facets, where the lattice fluctuations were revealed by PDF analysis.^[^ ^262^ ^]^ When the ratio of Pt/Ni reaches to 3/2, the lattice of Pt‐Ni nanowires expands for a significant lattice strain, which explains the optimized electrocatalytic activity of ORR. A similar work probed the surface lattice strain of twisty nanowire (TNW) shaped PtFe alloy with variable bimetallic compositions.^[^ ^263^ ^]^ By the X‐ray PDF analysis, the PtFe TNWs show a two‐phase structure mixing with *fcc* and *bcc* lattice with remarkable lattice strain. This lattice strain coupled with specific facets are supposed to be responsible for the enhanced electrocatalytic activity and durability.

The phase and compositions in the bimetallic nano‐electrocatalysts usually segregate on the surface of particles. These segregations surely affect the electrocatalytic activity and deserve to be investigated.^[^ ^264^ ^]^ In this respect, the PDF method coupled with big‐box RMC simulation might be one of the only choices to offer statistic view of the surface segregation. For example, the structural and compositional evolutions of Pt‐Ni alloys during electrocatalysis were revealed by the in situ PDF analysis.^[^ ^265^ ^]^ For the pristine PtNi_6_ nanoparticles, the Pt atoms are randomly distributed inside a face‐centered cubic matrix of Ni atoms. Upon electrochemical cycling, the Ni atoms leach from the surface of particles, leading to a chemically ordered Pt‐rich phase that is similar with the Au_3_Cu alloy. By contrast, the Ni atoms on the surface of PtNi_3_ nanoalloy are leached more quickly and a PtNi‐core/Pt‐shell architecture is formed. This will induce compressive surface strain that accounts for the enhanced catalytic activity. Besides, the atomic structures of Pd*_n_*Cu_100‐_ *_n_* nanoalloys with different compositions are characterized by X‐ray PDF method.^[^ ^266^, ^267^ ^]^ The PDF refinement results indicate that the Pd_50_Cu_50_ nanoparticles are fingerprinted by a mixed phase composed of chemically ordered *bcc* and disordered *fcc* domains, which turns to a single phase with potential cycling (Figure 12b). This structural change, triggered by the surface dissolution of Cu atoms, is demonstrated to be responsible for the enhanced ORR activity. Furthermore, Prasai et al. determined the atomic structures of Pt‐Ru alloy nanoparticles with the RMC simulations on *G*(*r*) patterns.^[^ ^268^ ^]^ In this way, the 3D structural models for the Pt*_x_*Ru_100‐_ *_x_* (*x* = 31, 49, 75) alloy nanoparticles could be extracted from the RMC simulation, where the compositional segregations could clearly visualized.

Apart from the above, the synthetic processes of the electrocatalytic nanoalloys, which is an important topic in this research field, could be tracked in terms of time‐resolved in situ PDF measurements. For example, the formation kinetics of Pt nanoparticles as a function of sintering temperatures was investigated by differential PDF (DPDF) technique under in situ condition.^[^ ^269^ ^]^ The evolutions of Pt‐Cl and Pt‐Pt correlations for both precursors and nanoparticles during the synthetic process were clearly revealed. Furthermore, the structural changes of PtNi nanoalloys supported on hollow graphitic spheres (HGSs) were probed during the synthesis route, in order to reveal the structural order/disorder phenomena.^[^ ^270^ ^]^ The results show that a disordered PtNi alloy could be formed upon rapid heating, whereas a disorder–order transition occurs under slow heating‐cooling procedure (Figure 12c).

On the other hand, the PDF technique also plays an important role in determining the local structures of other type of heterogeneous catalysts, for which the activity and selectivity of these catalysts can be optimized. For example, the structural changes of 4 w% Pd/Al_2_O_3_ during the oxidation and reduction processes were investigated by the in situ PDF measurements.^[^ ^271^ ^]^ Based on the PDF refinements with confined multi‐cluster models, the PdO‐Pd phase transition at elevated temperatures has been quantificationally analyzed. This provides a deeper insight into the structural‐catalytic mechanism in this material. In addition, the phase composition as well as the local structure of Pt/*γ*‐Al_2_O_3_ catalysts were studied by PDF method.^[^ ^272^ ^]^ It is found that the *γ*‐Al_2_O_3_ support possesses a large number of Al vacancies, and the Pt ions reside in the defects at octahedral sites. These ionic platinum centers at defective Al sites show higher selectivity in the model reaction of *n*‐heptane aromatization. In another case, Shan and co‐workers investigated the atomic structure of PdNi nanoalloys for establishing the structural‐catalytic synergy during the CO oxidation reaction.^[^ ^273^ ^]^ The 3D model of atomic structure is extracted from the RMC simulation of PDF data, by which the phases, compositions, and atomic arrangements over one PdNi nanoparticle could be revealed. These structural details show a strong interplay with the catalytic activity of CO oxidation. Furthermore, the in situ PDF measurements at variable temperatures were conducted to investigate the structural interactions between water and Brønsted acidic sites in the SAPO‐34 catalyst.^[^ ^274^ ^]^ The PDF analysis shows that the Al—O bond lengths exhibit a sudden change in the 250–300 °C region, which is related closely to the increased proton mobility in this catalyst (Figure 12d). Sommariva et al. performed PDF measurement to study the nano‐scale PdO catalyst supported on *γ*‐Fe_2_O_3_.^[^ ^275^ ^]^ A two‐phase structural model consisting of PdO and *γ*‐Fe_2_O_3_ phases is employed to describe the *G*(*r*) pattern, which confirms the co‐existence of these two phases in this hybrid catalyst. Within the catalytic dynamics, the structural changes of Al_2_O_3_‐supported Pt catalyst during the adsorption of H_2_ and CO were probed by in situ PDF measurements.^[^ ^276^ ^]^ The results show that the Pt—Pt bond lengths contract at the surface of particles. Upon the adsorption of CO and H_2_, this contraction is relaxed for surface reconstruction toward bulk‐like *fcc* phase, which is correlated with the catalytic activity.

Furthermore, the PDF method has been also frequently used to study the nanostructures of photocatalysts. For example, Thorsten et al. investigated the structures of photocatalyst Bi_2_WO_6_ at various sizes, which strongly couples the spectroscopic features.^[^ ^277^ ^]^ By using the PDF, the local geometries of BiO*_x_* and WO_6_ polyhedrons as well as their deformation in quantum‐dot‐sized Bi_2_WO_6_ have been clearly clarified. In another case, the nanostructure of hydrothermally prepared BiEuWO_6_ and BiTbWO_6_ photocatalysts have been encoded by neutron PDF coupled with long‐range probes such as synchrotron and neutron XRD refinements.^[^ ^278^ ^]^ In this way, the interplay between local structure and photocatalytic activity has been explored. Subsequently, the local bonding environments of Sillén–Aurvillius structured Bi_4_TaO_8_Cl nano‐photocatalyst have been probed by neutron PDF analysis.^[^ ^279^ ^]^ Compared with the bulk Bi_4_TaO_8_Cl, the local structure of the nano‐ Bi_4_TaO_8_Cl exhibits obvious deviations. The results show that the TaO_6_ octahedral distortion increases accompanied with a decrease of Ta—O—Ta bond angle for the nano material, which impacts the band structure and electron‐hole migration. Moreover, synchrotron PDF analysis was also employed to study the local structure of NH_3_‐loaded Fe(II)‐hydroxy‐phosphonoacetate substituted with Mn(II) isomorphic dopants (M = Mn, Co, and Zn).^[^ ^280^ ^]^ The PDF results confirm the loss of crystallinity upon NH_3_ adsorption, but the short‐range ordering could be maintained. Accordingly, the relationship between the improved photocatalytic performance and the local structural changes is clarified. Furthermore, in situ PDF was carried out to investigate the local structural changes of graphene oxide (GO)/TiO_2_ composites under H_2_ reduction.^[^ ^281^ ^]^ The evolutions of Ti—O and Ti—Ti bonds arising from hydrogenation are clearly identified, which evidences the C—C and Ti—C/Ti‐H contributions under Ti—O transition. This work helps to understand the relationship between the dynamic structural transition and a highly active photocatalyst during the synthesis process.

### 3.6 Energy Storage Materials

Rechargeable batteries, powering the majority of portable electronics and electric vehicles (EVs), have been a research hotspot in today's highly mobile society.^[^ ^282^ ^]^ Typically, the battery systems consist of four different components: cathode, anode, electrolyte, and separator. Although every single component is essential, it is the electrode material (i.e., cathode and anode) that determines the capacity and cut‐off voltage of a battery, and the electrochemical performance of the electrode materials is strongly coupled with the multi‐scale structures from long‐range to short‐range levels.^[^ ^16^ ^]^ For most of the electrode materials, the long‐range average structures and their evolutions upon changing and discharging have been clarified by in situ XRD measurements. Hence, the current research focus of electrode materials has been shifted gradually to the short‐range local structures, with the efforts being paid on two aspects: i) The design and optimization of static local structures. In order for desired performance, the cathode materials are usually designed with defects and cation disorders, or integrated with dopants, while the anode materials are frequently made of nanoscale, nanostructured or even amorphous materials. The success of these efforts relies on an in‐depth understanding of the short‐range local structure. ii) Tracking the local structural evolution during electrochemical process. Upon charging and discharging, the repetitive (de)intercalations of alkali‐metal ions are actually inhomogeneous, thus inducing distorted or even amorphous structures that are undetectable for the commonly used in situ XRD. With this regard, the PDF method that is complementary to the conventional diffraction‐based crystallography deserves to be undertaken in the researches of energy storage materials.

#### 3.6.1 Static Local Structure in As‐Prepared Electrode Materials

Layered TM oxides, that is, LiNi*_x_*Co*_y_*Mn_1‐_ *_x_*_‐_ *_y_*O_2_, (referred as NCM), are regarded as the leading candidates of cathode materials for lithium ion batteries (LIBs).^[^ ^283^ ^]^ For these materials, the cation disorders in the TM layers are supposed to play an important role in Li^+^ diffusion kinetics. In this respect, a series of PDF investigations on the ordering of TM cations were carried out by Grey and co‐workers.^[^ ^284^, ^285^, ^286^ ^]^ These studies have distinguished different TM—O bond lengths by the neutron PDF analysis. In addition, the RMC simulations reveal a non‐random distribution of the TM cations in Li[Ni_0.5_Mn_0.5_]O_2_, while the Li[Ni_1/3_Mn_1/3_Co_1/3_]O_2_ cathode shows a less‐random distribution of Ni and Mn and a more randomly distributed Co in TM layers. Besides, the PDF method shows that the lithium transportation dynamics in layered Li‐rich materials could be affected by the TM distributions.^[^ ^287^, ^288^ ^]^ Furthermore, the PDF measurements were also employed to reveal the short‐range ordering in the rock‐salt‐type Li‐rich materials, which offers a deeper understand of the structure‐performance coupling. For example, the neutron PDF measurements were carried out to clarify the local structural impacts on the performance of two Li‐rich materials—Li_1.2_Mn_0.4_Zr_0.4_O_2_ (LMZO) and Li_1.2_Mn_0.4_Ti_0.4_O_2_ (LMTO).^[^ ^289^ ^]^ Apart from the LIB cathodes, the PDF technique has been also frequently used to reveal the short‐range ordering in layered oxide cathode materials for sodium‐ion batteries.^[^ ^290^, ^291^ ^]^ Different from the average structure of NaNi_2/3_Sb_1/3_O_2_ revealed by XRD, the PDF results suggest that the material possesses a honeycomb structure similar with Li‐/Na‐rich materials, indicating the emergence of short‐range ordering.

Different from the cathode materials, the anode materials are typically made of amorphous or nanostructured carbon or silicon, so their electrochemical performance is strongly coupled with short‐range atomic structures. By using the PDF method, the local bonding environments in the carbon materials can be quantitatively characterized. For example, the PDF measurements were conducted on a porous carbon material (OSPC).^[^ ^292^ ^]^ The PDF patterns show an additional peak corresponding to the C—C bonds with hybridzed *sp* and *sp* ^3^ orbitals, which is different from the conventional carbon materials. In addition, Li et al. reported that the hard carbon treated with microwave shows a high performance superior to the heating‐treated carbon.^[^ ^293^ ^]^ From their PDF profiles, the hard carbon possesses lower amplitude of peaks, indicating a high extent of structural vacancies that are responsible for a slope capacity. Furthermore, the Si‐based materials, as a class of high‐performance anode materials, have been also investigated by the PDF analysis. For example, the Si nanoparticles with different particle sizes were synthesized for electrochemical testing. The PDF analysis was carried out to probe the degree of crystalline and local bonding geometry for further mechanism investigation.^[^ ^294^ ^]^

#### 3.6.2 Dynamically Evolved Local Structure under Battery Operation

##### Ex Situ PDF Measurements for Studying Charged/Discharged Battery Materials

The characterization of local structural dynamics upon charging and discharging is an important but challenging research area in the field of energy storage materials. In general, the best way to track these local structural changes is to carry out an in situ PDF measurement, but the realization is always a challenging task for the battery researches, since the cell components out of interests (such as carbon, electrolyte, counter electrode) also generate strong scattering signals. The compromise solution for this challenge is to pre‐charge (or pre‐discharge) the electrode to a certain voltage state, then to disassemble the cell and scrape the powders from electrode for ex situ PDF measurement.

**Figure 13:**
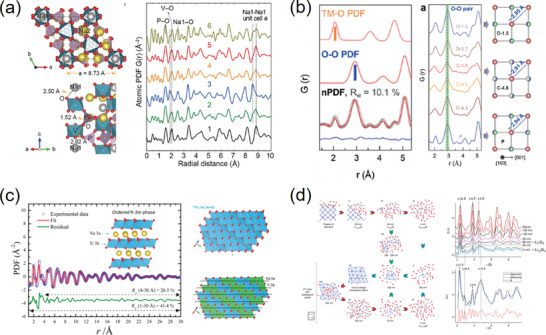
a) The PDF analysis for Na_3_V_2_(PO_4_)_3_ samples at various charge states. Reproduced with permission.^[^ ^298^ ^]^ Copyright 2017, Elsevier. b) The neutron PDF patterns (left) and the variation of average O‐O distance (right). Reproduced with permission.^[^ ^300^ ^]^ Copyright 2019, Wiley. c) The PDF refinement of the fully discharged Na*_x_*TiO_2_ with O3‐type structure model (space group: *R*‐3*m*). Reproduced with permission.^[^ ^302^ ^]^ Copyright 2017, American Chemical Society. d) Illustration of mechanisms by which silicon is lithiated and delithiated, and the ex situ PDF and fitting of battery samples collected during the first discharge. Reproduced with permission.^[^ ^304^ ^]^ Copyright 2011, American Chemical Society.

For example, Talaie et al. elucidated the high‐voltage structure of P2‐Na_0.67‐_ *_z_*Fe_0.5_Mn_0.5_O_2_ (*z* ≈0.5) cathode by the X‐ray PDF measurement.^[^ ^295^ ^]^ The results show that the Fe^3+^ ions migrate into interlayer tetrahedral sites at high potential. These Fe^3+^ ions residing between the TM layers could act as barriers for hindered Li‐ion transportation, which explains the large polarization and performance degradation in this material. In addition, the X‐ray PDF was also used to study the atomic structural changes of MOF cathode material (Fe‐MIL‐100) for Na‐ion battery.^[^ ^296^ ^]^ By means of PDF refinement, the capacity decay in this material is revealed to be derived from the inaccessibility of active sites in the cathode material for Na ions. Moreover, the local structural changes in olivine NaFePO_4_ was investigated by X‐ray PDF method.^[^ ^297^ ^]^ In this way, the crystalline‐amorphous transition upon deeply charging is clarified, suggesting a new strain‐accommodation mechanism occurred in the charged samples. In a similar case, Li and his co‐workers detected the local structural changes of Na_3_V_2_(PO_4_)_3_ cathode by the PDF analysis.^[^ ^298^ ^]^ The PDF results show that the lattice expansion along *ɑ* direction is related to the change of V—O bond lengths during cycling, which is responsible for the high performance of this material (**Figure** **13**a). In addition, Li‐rich or Na‐rich cathode materials have gained extensive attentions due to their exceptionally high capacity realized by the anion redox reactions in high voltage. Here, the X‐ray PDF was carried out by Hong et al. to study the structure‐related oxygen‐redox activity of Li_2‐_ *_x_*Ir_1‐_ *_y_*Sn*_y_*O_3_.^[^ ^299^ ^]^ The results indicate that the shortened O—O dimers could trigger the oxygen redox reaction at high voltage.

The cathode materials usually contain light atoms (like Li) and nearby TM elements (e.g., Mn, Ni, and Co). Since the X‐ray‐based techniques show difficulty to distinguish these atoms accurately, the neutron PDF technique, which is complementary to the X‐rays, plays an irreplaceable role in studying energy storage materials. For example, Zeng et al. employed the neutron PDF coupled with RMC simulations to probe the local Ni/Co/Mn ordering in the NCM111 cathode.^[^ ^288^ ^]^ It is revealed that the atomic arrangements of TMs greatly affect the Li‐diffusion kinetics and show a strong impact on the electrochemical performance in the NCM111 material. In addition, Zhao et al. employed neutron PDF to investigate the structure‐correlated oxygen‐redox behavior in the Li_1.2_Ti_0.35_Ni_0.35_Nb_0.1_O_1.8_F_0.2_ Li‐rich cathode. The results show that the Li/TM cation orders play an important role in the oxygen redox reactivity (Figure 13b).^[^ ^300^ ^]^

Compared with cathode materials, the structure transitions in anode materials are more complicated. Yamakawa et al. investigated the local structural changes of FeF_3_ at different discharged states using the X‐ray PDF technique.^[^ ^301^ ^]^ The resulting PDF data indicates that the reconversion reaction occurs via the reformation of lithiated rutile phase. This could provide the theoretical basis for the enhanced reversibility and rate capability of binary TM compounds. Furthermore, the atomic structures of pristine, fully discharged and fully re‐charged TiO_2_ nanoparticles for Na‐battery anode were probed by the ex situ PDF measurements (Figure 13c), suggesting that the Na ions could irreversibly insert into the interstitial sites. This will induce the loss of structural ordering and thereby result in severe performance degradation.^[^ ^302^ ^]^ In a similar case, the anatase TiO_2_ nanoparticles with Ti‐vacancy were prepared for the use of Na‐ion battery anode and studied by X‐ray PDF technique.^[^ ^303^ ^]^ By comparing the structures of as‐prepared and discharged samples, the Na ions tend to embed into the Ti vacancies, revealing the sodium storage mechanism of defective TiO_2_.

Another important class of anode materials for Li‐ or Na‐ion batteries is that of the simple substance of metals (e.g., Fe) and non‐metals. (e.g., C and P). Key et al. employed X‐ray PDF to study the structural evolution of Si anode during charging and discharging processes (Figure 13d).^[^ ^304^ ^]^ It is revealed that the lithiation of crystalline silicon undergoes a complex process. It starts from bonding breakage of Si matrix and then forms small clusters until the bulk crystalline Si is consumed and totally amorphized. In addition, Jung et al. investigated the detailed structural transitions of Ge anode by X‐ray PDF technique.^[^ ^305^ ^]^ The PDF results show that the crystalline Ge gradually transforms to Li_15_Ge_4_ alloy via the breakage of Ge—Ge bond. Sottmann et al. employed amorphous phosphorus (P) to be used as the anode for Na‐ion battery. Through the X‐ray PDF characterization, the structural evolution mechanisms were investigated in the sodiation and desodiation process, where the as‐prepared amorphous P anode transforms to a crystalline Na_3_P phase when fully discharged.^[^ ^306^ ^]^

##### In Situ PDF Measurement for Operating Battery

**Figure 14:**
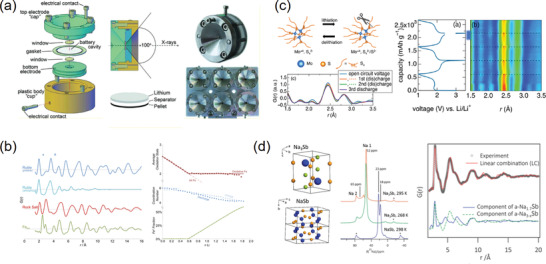
a) Schematic diagram and photo of the AMPIX cell for in situ PDF measurement. Reproduced with permission.^[^ ^307^ ^]^ Copyright 2012, International Union of Crystallography. b) Simulated PDFs corresponding to the pristine rutile, rock salt, Fe nanoparticles, and amorphous rutile phase formed within the electrode during cycling. Reproduced with permission.^[^ ^308^ ^]^ Copyright 2013, American Chemical Society. c) Schematic of the polysulfide chain scission in molybdenum chalcogels upon discharge and/or lithiation, and the contour plot of in situ PDF data along with charge–discharge curves. Reproduced with permission.^[^ ^310^ ^]^ Copyright 2016, American Chemical Society. d) Unit cell of Na_3_Sb and 1 × 2 × 1 unit cells of NaSb showing the helical chains of antimony, and results of linear combination fitting of the PDFs extracted during processes S2‐b and S2‐c with PDFs for a‐Na_1.7_Sb and a‐Na_3–_ *_x_*Sb. Reproduced with permission.^[^ ^311^ ^]^ Copyright 2016, American Chemical Society.

The capability of probing batteries under operando condition shows significant advantage over the ex situ measurements. For example, the ex situ approach not only limits the voltage states to be studied, but also increases the risks of charging‐state relaxation, short circuits, and moist deterioration during cell disassembly. In contrast, in situ PDF characterization can effectively address the above issues, and it is able to capture the transient states with high accuracy. However, conducting in situ PDF measurements on a running battery is a great challenge. In practice, the most challenging task for collecting reliable in situ data is how to subtract the scattering intensities out of interests, such as carbon black, Li or Na metal, current collector, and so on. The common approach is to reproduce these signals as backgrounds, but the strong diffraction texture generated from Al or Cu current collectors cannot be avoided based on conventional coin‐cell measurements. Additionally, in order to collect scattering data over a broad *Q*‐range, the electrode must be allowed to reach over a wide scattering angular range. These challenges put forward stringent requirements on the design of in situ battery devices. The Argonne's multi‐purpose in‐situ X‐ray (AMPIX) cell, consisting of a cup‐shaped body, two tablet electrodes at the top and bottom, two X‐ray transmissive windows, and a flat annular gasket that is sandwiched between the electrodes, is the best choice for in situ X‐ray PDF measurements (**Figure** **14**a).^[^ ^307^ ^]^ This cell is free of current collectors, and enables a maximum prevention of signals that come from the cell components out of interests. Based on this AMPIX cell, some progress has been made in probing the local structural dynamics with in situ PDF technique.

For example, the local structural evolution of the Fe^II^_(1‐_ *_x_*_)_Fe^III^ *_x_*O*_x_*F_2‐_ *_x_* (*x* = 0–1) anode material was investigated by in situ PDF experiments (Figure 14b).^[^ ^308^ ^]^ Although the discharge capacity could be recovered upon charging, the atomic structure for the cycled electrode differs from the pristine state, which finally transforms to a mixed phase consisting of amorphous rutile nanoscale rock salt phases. In addition, the local structural changes of RuO_2_ during the electrochemical process was studied by in‐situ PDF analysis.^[^ ^309^ ^]^ The results show that extra capacity in this system is derived from the generation of LiOH and its subsequent reversible reaction with Li to form Li_2_O and LiH. In general, sulfur cathodes in LIBs can deliver a high gravimetric capacity but suffer from parasitic polysulfide shuttling. This harmful shuttling effect could be suppressed in a TM chalcogels of MoS_3.4_.^[^ ^310^ ^]^ The in‐situ PDF study demonstrated the transform of S—S bonds and maintained Mo—S bonds during cycling, indicating that the electrochemical reversibility of these high‐capacity chalcogel electrodes is attributed to their amorphous structures (Figure 14c). Moreover, the in situ PDF technique could be also employed to gain insight into the alloying mechanism of high‐capacity antimony anodes (Na*_x_*Sb).^[^ ^311^ ^]^ The short‐range structural evolution is revealed to couple a series of sequential Na‐reactions under different voltages (Figure 14d).

## 4 Summary and Perspectives

In this review, we have first summarized the basic principles, data analysis methods, and unique capabilities of the PDF technique, followed by practical applications in a variety of functional materials. As the importance of structural complexity has been gradually realized by the functional materials community, the PDF technique is becoming widely adopted. A good example of this might be the PDF characterizations in under‐developing “non‐ideal” metal‐organic framework (MOF) materials, which are embedded with highly disordered structure and unpredictable novel properties relevant to conductivity and catalysis.^[^ ^312^ ^]^ In addition, recent advances in the total magnetic scattering (i.e., mPDF), on account of the interaction between neutrons and magnetic moments of atoms,^[^ ^313^, ^314^ ^]^ have enabled to reveal a short‐range aligned magnetic order. With the theoretical equation being derived in 2014,^[^ ^315^ ^]^ the mPDF method has led to deeper insights into the materials with complex magnetic structures such as spin‐liquid Gd_3_Ga_5_O_12_,^[^ ^316^ ^]^ ground‐state MnO,^[^ ^317^ ^]^ and so on. Within new technologies, the PDF method is expected to be possibly conducted with an X‐ray free‐electron laser oscillator (XFELO) in the near future. The XFELO yields 4 orders of magnitude higher flux (7 orders in brightness) than the current third‐generation synchrotron facilities,^[^ ^318^ ^]^ so the speed in PDF data collection will be promoted to a large extent. Furthermore, the PDF technique is also developed to couple with computed tomography. In this way, the structural inhomogeneities inside the objects or devices, for example the commercial Ni/MH battery,^[^ ^319^ ^]^ could be directly visualized.

Despite the rapid promotion, nevertheless, there are still limitations that are needed to be addressed. The conventional PDF method is element insensitive, which is a major disadvantage relative to the EXAFS approach. This drawback has been overcome by the recent development of element‐specific DPDF technology. It is extracted from the so‐called coherent XRD, making use of the anomalous dispersion of X‐rays close to the absorption edge of a certain element.^[^ ^320^ ^]^ Analogous to the EXAFS, the DPDF pattern only consists of the atom‐pair correlations regarding the element whose absorption edge is detected. Based on this, the elements with similar X‐ray or neutron scattering capabilities can be clearly separated, which has ignited advances in the fields of ferroelectrics,^[^ ^321^ ^]^ catalysts,^[^ ^322^, ^323^ ^]^ semiconductors,^[^ ^324^ ^]^ and so on. On the other hand, it is still a great challenge to conduct PDF experiments under operando conditions. Especially, the measurement of in situ PDF for battery materials is the most difficult one, since the battery cell components that are out of interests (i.e., carbon black, electrolyte, binder, and so on) also generate significant scattering signals. Therefore, the design of in situ cells is always the primary concern for the in situ battery experiments. As mentioned above, the AMPIX cells are adopted widely for the in situ PDF of X‐ray total scatterings, and novel battery design includes diamond radial in situ X‐ray (DRIX) cell for fast operando data collection.^[^ ^325^ ^]^ For the PDF of neutron scattering, the realization of in situ condition has rarely been reported. Since knowledge of light‐element dynamics (e.g., Li‐ion transportation, oxygen sublattice distortion, and so on) has not been clearly clarified for many battery materials, it is of great significance to develop an in situ neutron PDF technique for battery material investigations.

## Conflict of Interest

The authors declare no conflict of interest.
